# Wheat-Surface Microbiota as a Key Driver of Daqu Quality: Evidence from Microbial Diversity and Volatile Flavor Analysis

**DOI:** 10.3390/foods15193511

**Published:** 2026-10-01

**Authors:** Ying Huang, Huan Guo, Hao Liu, Fang Wei, Hong Deng, Hong Zhang, Li Jiang, Yu Mu

**Affiliations:** 1 School of Brewing Engineering, Moutai Institute, Renhuai 564507, China; huangying12349@163.com (Y.H.); molihao1006@163.com (H.L.); 17586026535@163.com (F.W.); 2 School of Food Engineering, Moutai Institute, Renhuai 564507, China; 19882595863@163.com (H.G.); hongzhang1986@163.com (H.Z.); 3 School of Resources and Environment Engineering, Moutai Institute, Renhuai 564507, China; dengh19980221@163.com; 4 School of Liquor and Food Engineering, Guizhou University, Guiyang 550025, China

**Keywords:** Daqu, wheat-derived microorganisms, microbial community, Niche analysis, volatiles, correlation analysis

## Abstract

Traditional fermentation processes rely on complex microbial communities; however, the specific contributions of raw material-associated microorganisms to community assembly and flavor formation remain unclear. In this study, traditional Daqu (T) and surface-disinfected Daqu (S) were used as paired controls to decouple the contribution of raw material-associated microorganisms. By integrating community, flavor, and functional analyses, we revealed their ecological roles in driving deterministic assembly, reshaping fungal interactions, and associating with characteristic flavors. The results showed that T was dominated by *Saccharopolyspora* and *Thermoascus*, whereas S was dominated by mesophilic lactic acid bacteria such as Weissella. The selective pressure imposed by wheat-associated microorganisms drove deterministic community assembly and shifted fungal interactions from competition to positive mutualism. A total of 110 flavor compounds were identified, from which 21 potential contributing compounds and 25 differential markers were screened. The characteristic flavors of T were tetramethylpyrazine, dimethyl trisulfide, benzaldehyde, isovaleric acid, and 2-methoxy-4-vinylphenol, whereas those of S were mainly 1-octen-3-ol, 2-octanone, and phenylethyl alcohol. Functional prediction and correlation analysis showed that wheat-associated microorganisms were positively correlated with primary metabolic pathways such as those involving amylase and alcohol dehydrogenase, while *Saccharopolyspora*, *Cutaneotrichosporon*, and specific *Bacillus* species were positively correlated with secondary metabolism and characteristic flavor formation. This study deepens the understanding of the ecological roles of raw material-associated microorganisms and provides a scientific basis for process optimization and microbial community regulation in traditional fermented foods.

## 1. Introduction

Daqu is a key saccharifying and fermenting starter in Baijiu production. Its microbial community, enzymatic potential, and metabolic activity play important roles in determining fermentation performance and flavor formation [1,2,3]. High-temperature Daqu is a typical type, usually using wheat as the main raw material. Its production includes wheat moistening, crushing, water addition, pressing into blocks, and controlled fermentation. After molding, the product temperature rises rapidly, reaching a maximum of about 60 °C, and then gradually decreases until the end of fermentation. This high-temperature stage imposes strong selective pressure on the microbiota. Many mesophilic vegetative cells and non-thermotolerant fungi associated with wheat may be inhibited or inactivated, whereas thermotolerant spore-forming bacteria and thermophilic fungi may survive and even proliferate. Traditionally, Daqu microorganisms have been considered to originate mainly from the production environment, including workshop air and equipment-associated microorganisms [4,5]. In comparison, the microbial community carried by wheat raw materials may be an important source of the early-stage community in high-temperature Daqu fermentation.

Recent studies have shown that wheat-associated microorganisms such as *Staphylococcus*, *Pantoea*, *Alternaria*, and *Mycosphaerella* are abundant in the early stage of high-temperature Daqu fermentation, whereas environmental or fermentation-adapted taxa such as *Bacillus* and *Thermoascus* become more abundant in the later stage [6]. In addition, Daqu-derived microorganisms also contribute substantially to the subsequent Baijiu fermentation microbiota [7]. However, it remains unclear how much wheat-associated and environmental microorganisms contribute to community succession, final community composition, and flavor quality of Daqu. Some studies emphasize the importance of raw-material microbiota in early community assembly, whereas others highlight the effects of environmental heterogeneity, including spatial position, temperature, and humidity, on microbial networks and flavor metabolism [8,9].

Although wheat-associated microorganisms are considered to play important roles in high-temperature Daqu fermentation, direct experimental evidence for their contribution to community succession and flavor formation remains limited [10,11]. Most previous studies relied only on source tracking or correlation-based methods, which cannot fully distinguish the contributions of wheat-associated microorganisms from those of the surrounding environment [12]. Therefore, controlled comparison by reducing raw-material microorganisms is an effective strategy to separate their contributions; however, whether these early microbial differences can persistently affect key functional taxa and the formation of characteristic flavor compounds still needs to be verified by controlled experiments. In high-temperature Daqu fermentation, clarifying this issue is important for moving from natural inoculation to precise microbial regulation.

In this study, we established two high-temperature Daqu fermentation systems using untreated and surface-disinfected wheat, thereby decoupling the contribution of raw-material-associated microorganisms from the environmental background through a controlled comparison. We integrated amplicon sequencing, headspace solid-phase microextraction gas chromatography-mass spectrometry (HS-SPME-GC-MS), community assembly analysis, co-occurrence network analysis, functional prediction, and microbe-metabolite correlation analysis to evaluate the contribution of wheat-associated microorganisms to Daqu fermentation. Specifically, this study aimed to: (1) characterize their effects on bacterial and fungal succession and community assembly; (2) determine their effects on volatile flavor profiles and key aroma-active compounds; and (3) identify microbial taxa associated with characteristic flavor compounds. These findings not only deepen our understanding of the role of raw-material microbiota in high-temperature Daqu fermentation but also provide a basis for raw material pretreatment, targeted supplementation of key functional microorganisms, development of fortified starters, and optimization of the Daqu-making process, thereby improving the quality stability of Daqu and the consistency of Baijiu production.

## 2. Materials and Methods

### 2.1. Experimental Design and Sample Collection

Wheat used for high-temperature Daqu production was obtained from a sauce-flavor Baijiu manufacturer (Guizhou Tangzhuang Chinese Liquor Limited company, Zunyi, China). A total of 100 kg of wheat was divided equally by mass into two portions (50 kg each; 1:1 ratio). One portion was treated to reduce wheat-associated microorganisms by immersion in 75% ethanol (prepared in-house) for 15 s, followed by three rinses with sterile distilled water and ultraviolet irradiation for 30 min; this portion was used for the surface-disinfected group (S). The other portion (50 kg) was left untreated and used for traditional Daqu production (traditional group, T). Both wheat treatments were independently processed under identical high-temperature Daqu production conditions for 40 days. The product temperature was monitored at the center of the Daqu blocks and reached a maximum of 60 °C.

A total of 50 Daqu blocks were prepared: 25 in the T group and 25 in the S group. Each group (25 blocks) was processed as one independent fermentation batch. At each sampling point (days 0, 10, 20, and 40), four blocks were randomly selected from each group. Each block was thoroughly crushed and homogenized, and each block served as one representative sample. In total, 32 representative samples were obtained (2 groups × 4 time points × 4 blocks). All samples were transported to the laboratory on ice within 3 h and stored at −80 °C until further analysis. Four biological replicates were collected for each treatment at each sampling time. Samples were labeled T_d0, T_d10, T_d20, and T_d40 for group T, and S_d0, S_d10, S_d20, and S_d40 for group S.

### 2.2. Amplicon Sequencing

Approximately 7 g of ground Daqu sample was used for total genomic DNA extraction with the E.Z.N.A.^®^ Soil DNA Kit (Omega Bio-tek, Norcross, GA, USA) according to the manufacturer’s instructions. DNA integrity was evaluated by 1% agarose gel electrophoresis, and DNA concentration and purity were determined using a NanoDrop 2000 spectrophotometer (Thermo Scientific, Waltham, MA, USA). For bacterial community analysis, the V3–V4 region of the 16S rRNA gene was amplified using the primer pair 338F (5′-ACTCCTACGGGAGGCAGCAG-3′) and 806R (5′-GGACGGACTACHVGGGTWCTAAT-3′). For fungal community analysis, the ITS1 region was amplified using primers ITS1F (5′-CTTGGTCATTTAGAGGAAGTAA-3′) and ITS2R (5′-CCGTGTTTCAAGACGGG-3′). Sample-specific barcodes were incorporated into the primers for multiplex sequencing.

PCR amplification was performed in a T100 Thermal Cycler (Bio-Rad, Hercules, CA, USA) in a total reaction volume of 20 µL containing 4 µL of 5× TransStart FastPfu buffer, 2 µL of dNTPs (2.5 mM), 0.8 µL of each primer (5 µM), 0.4 µL of TransStart FastPfu DNA polymerase, approximately 10 ng of template DNA, and sterile water. The amplification conditions were as follows: initial denaturation at 98 °C for 3 min; 27 cycles of denaturation at 98 °C for 30 s, annealing at 55 °C for 45 s, and extension at 72 °C for 45 s; followed by a final extension at 72 °C for 5 min. PCR products were purified and quantified before paired-end sequencing on an Illumina NovaSeq platform by Shanghai Personal Biotechnology Co., Ltd. (Shanghai, China).

Raw sequencing reads were quality-filtered using fastp (v0.19.6) and merged using FLASH (v1.2.11). Reads were truncated when the average quality score within a 10 bp sliding window fell below 20. Reads shorter than 50 bp after quality filtering or containing ambiguous bases were discarded. Paired-end reads were merged with a minimum overlap of 10 bp and a maximum mismatch ratio of 0.2 within the overlapping region. Barcode sequences were required to match exactly, whereas up to two mismatches were allowed in primer sequences.

The resulting high-quality sequences were denoised using the DADA2 plugin implemented in QIIME 2 to generate amplicon sequence variants (ASVs) and remove chimeric sequences. Taxonomic assignment was subsequently performed, and sequences classified as chloroplasts or mitochondria were removed from the bacterial dataset. Samples were rarefied to an equal sequencing depth based on the minimum number of retained sequences among samples before downstream alpha- and beta-diversity analyses.

### 2.3. Analysis of Volatile Flavor Compounds

Volatile flavor compounds in Daqu samples were analyzed by HS-SPME-GC–MS (Agilent Technologies, Santa Clara, CA, USA), with modifications to the method described by Mu et al. [13]. Briefly, 3 g of ground Daqu sample was transferred into a 20 mL headspace vial, followed by the addition of 20 μL of methyl octanoate at a concentration of 0.0079 g/100 mL. The vial was immediately sealed and equilibrated at 60 °C for 5 min. A preconditioned 50/30 μm DVB/CAR/PDMS SPME fiber (Supelco, Bellefonte, PA, USA) was then exposed to the vial headspace at 60 °C for 45 min. After extraction, the fiber was immediately inserted into the GC injector for thermal desorption.

GC–MS analysis was performed using an Agilent 6890N gas chromatograph coupled to a 5975B mass selective detector (Agilent Technologies, Santa Clara, CA, USA) and equipped with a DB-WAX capillary column (60 m × 0.25 mm i.d., 0.25 μm film thickness). The injector temperature was maintained at 250 °C, and high-purity helium (>99.999%) was used as the carrier gas at a constant flow rate of 1.0 mL/min. Samples were injected in splitless mode. The oven temperature was initially maintained at 40 °C for 5 min, increased to 100 °C at 4 °C/min, and then increased to 230 °C at 6 °C/min and held for 10 min. The mass spectrometer was operated in electron ionization (EI) mode at 70 eV. The ion source and transfer line temperatures were maintained at 250 and 300 °C, respectively, and mass spectra were acquired over an m/z range of 35–400.

Volatile compounds were preliminarily identified by matching their mass spectra against the NIST mass spectral library, with a similarity threshold of ≥80%. Retention indices (RIs) were calculated using n-alkane homologues (C8–C40) analyzed under the same chromatographic conditions, and compared with reference RI values reported in the NIST WebBook and in the literature on DB-WAX or equivalent polar columns. Compounds with a NIST match of ≥80% and calculated RIs consistent with literature RI values were retained. All preliminarily identified compounds in this study met the Level 2 criteria, which indicates identification based on literature or database matching rather than confirmation with authentic standards. After identification, volatile compounds were semi-quantified using the internal standard method, and their relative concentrations were calculated according to the following formula:

C*_i_* = (A*_i_*/A*_IS_*) ×C*_IS_* (1)

where C*_i_* and A*_i_* denote the concentration (mg/L) and peak area of the analyte, and C*_IS_* and A*_IS_* denote the concentration and peak area of the internal standard.

### 2.4. Calculation of OAVs

To evaluate the potential contributions of volatile compounds to Daqu, we calculated the relative odor activity values (ROAVs) according to the method of Zhang et al. [14]. Compounds with ROAVs ≥ 1 may contribute to Daqu, whereas those with ROAVs < 1 are considered to have a minor contribution. The ROAVs were calculated using the following formula:

ROAVs = 100 × C*_i_*/C*_max_* × T*_max_*/T*_i_* (2)

where C is the relative content of volatile flavor substances, *T* is the threshold of the volatile flavor compounds, *i* refers to detected compounds and *max* is compounds with the maximum odour activity value.

### 2.5. Statistical Analysis

All experiments were performed with three biological replicates, and data are presented as the mean ± standard deviation (SD). Basic data processing and visualization were performed using Microsoft Excel and Origin 2021(Version 9.8). Differences in microbial community composition among samples were evaluated by principal coordinate analysis (PCoA) based on appropriate beta-diversity distance matrices. Random forest analysis was additionally performed to evaluate the relative importance of microbial taxa in discriminating among sample groups. Microbial community assembly processes were assessed using niche width analysis, the Sloan neutral community model, and the normalized stochasticity ratio (NST). An NST value of 0.5 was used as the threshold to distinguish predominantly stochastic (>0.5) from deterministic (<0.5) assembly processes. Microbial co-occurrence networks were constructed based on Spearman’s rank correlations (|r| > 0.7, *p* < 0.05) and visualized using Gephi (Version 0.11.3). Network-level topological properties, including network density and modularity, were calculated, and putative keystone taxa were identified based on within-module connectivity (Zi) and among-module connectivity (Pi). Differences in volatile flavor profiles were evaluated using partial least squares discriminant analysis (PLS-DA). Variable importance in projection (VIP) scores were calculated, and compounds with VIP > 1 and *p* < 0.05 were considered discriminant volatile compounds. The robustness of the PLS-DA model was assessed using 200 permutation tests. For bacterial and fungal communities, we used PICRUSt2 v2.2.2-b to perform functional prediction based on 16S rRNA and ITS feature sequences: feature sequences were aligned against reference sequences and a phylogenetic tree was constructed; the Castor hidden-state prediction algorithm was used to infer gene family copy numbers, and NSTI was calculated simultaneously; feature sequences with NSTI > 2.0 were removed. Subsequently, combined with the abundance of feature sequences in each sample, the gene family copy numbers of each sample were calculated, and a stratified strategy was used to retain the correspondence between functions and species. Finally, gene families were mapped to the MetaCyc database, and metabolic pathways were inferred using MinPath (Version 1.6) to obtain enzyme abundance data. Pearson correlation analysis was used to assess the correlations between core microbial genera and characteristic volatile compounds, and the results were visualized as heatmaps using the GenesCloud platform (https://www.genescloud.cn), a free online platform for data analysis. Statistical significance was defined as *p* < 0.05.

## 3. Results and Discussion

### 3.1. Microbial Diversity

**Figure 1:**
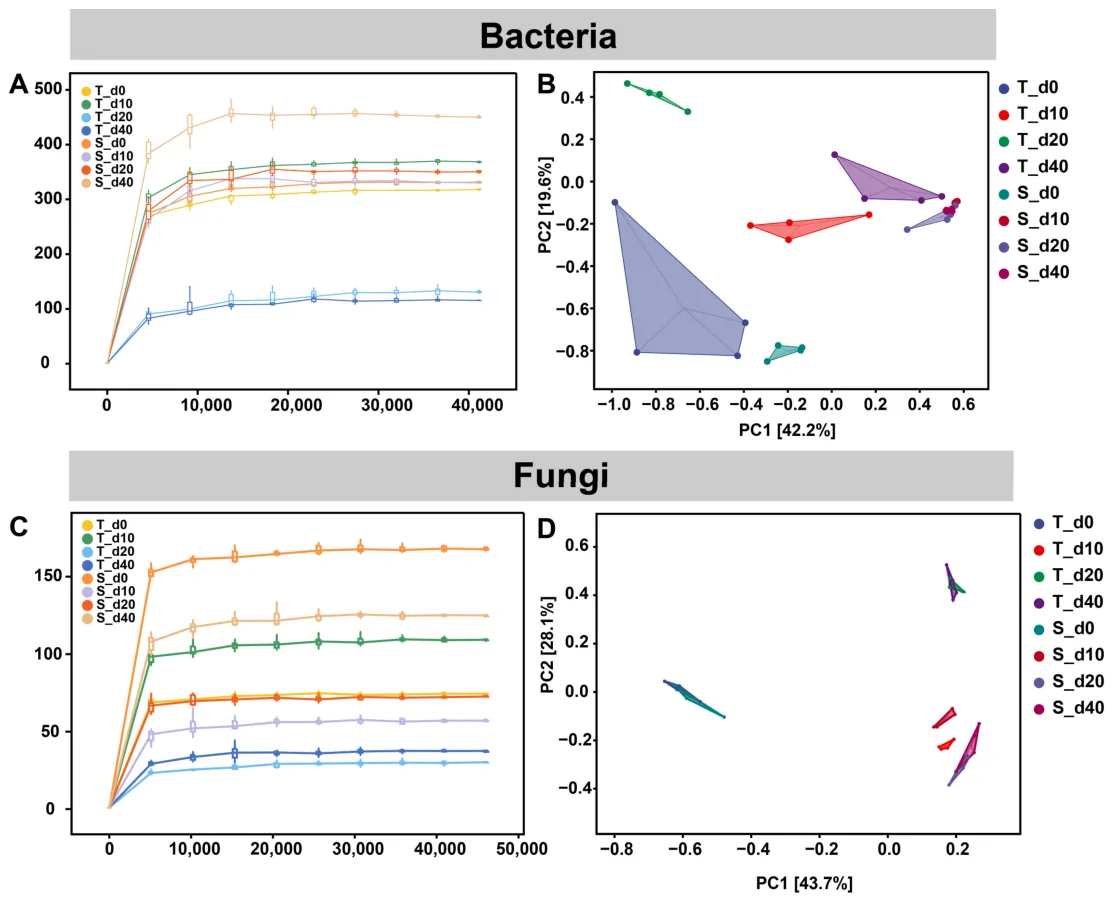
Dynamic changes in microbial diversity and community structure during Daqu fermentation. (**A**,**C**) Rarefaction curves of bacterial (**A**) and fungal (**C**) communities. (**B**,**D**) Principal component analysis (PCA) based on amplicon sequence variants (ASVs).

Rarefaction curves showed that the observed ASV numbers of both bacterial and fungal communities increased rapidly with sequencing depth and gradually approached a plateau beyond approximately 10,000 sequences, indicating sufficient sequencing coverage of microbial diversity (Figure 1A,C). PCA revealed clear separation of bacterial and fungal communities between groups T and S (Figure 1B,D), indicating that reduction in wheat-associated microorganisms altered the composition and successional trajectories of the Daqu microbiota [1]. Both groups showed greater community shifts during early fermentation, followed by increasing clustering at the mid-to-late stages. Fungal communities were more tightly clustered at 20 and 40 d, suggesting stronger compositional convergence during later fermentation [2].

Alpha-diversity analysis showed that bacterial diversity in group T increased from 0 to 10 d and then gradually declined, whereas group S exhibited an overall increasing trend from 0 to 40 d (Figure S1A–D). These contrasting patterns may be related to differences in the initial microbiota and subsequent colonization and environmental selection [3]. In group T, the coexistence of microorganisms from multiple sources may have increased early community diversity, whereas progressive enrichment of better-adapted taxa likely reduced diversity as fermentation proceeded [15]. In contrast, reduction in the initial wheat-associated microbiota in group S may have created more opportunities for subsequent colonization by exogenous microorganisms [16].

Fungal communities exhibited a different succession pattern (Figure S1E–H). Fungal diversity was higher in group S than in group T at 0 d, but decreased markedly in both groups at 20 d and slightly recovered at 40 d. This pattern was consistent with the high-temperature and low-moisture conditions typically occurring during the middle stage of high-temperature Daqu fermentation [17]. These results suggest that environmental filtering may become increasingly important during the mid-to-late stages, favoring thermotolerant and low-water-activity-tolerant fungi and promoting convergence of fungal communities between the two groups [18,19,20].

### 3.2. Core Microbial Composition and Differential Taxa

**Figure 2:**
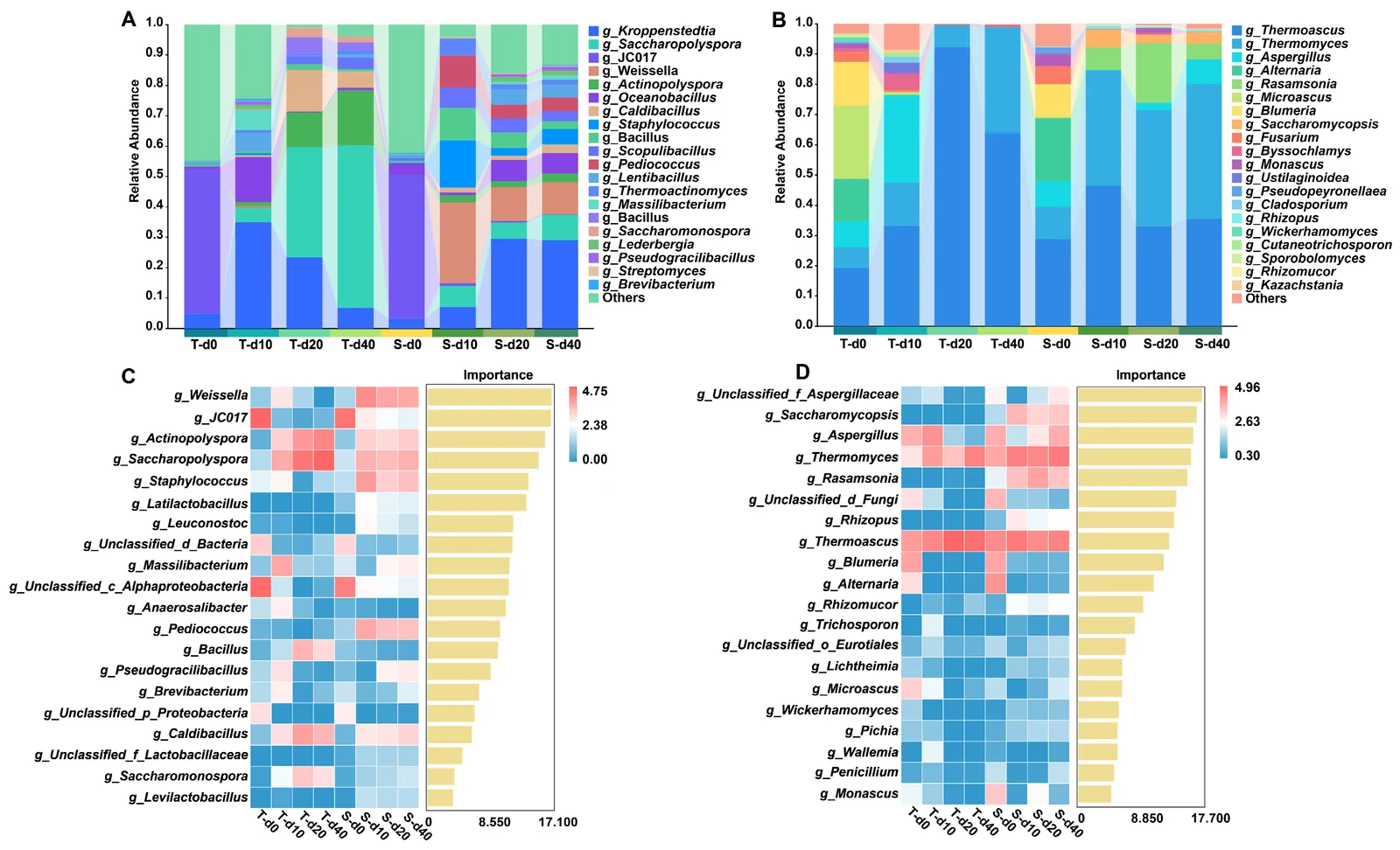
Microbial community composition and identification of key differential genera during Daqu fermentation. (**A**,**B**) Stacked bar plots showing the relative abundances of bacterial (**A**) and fungal (**B**) communities at the genus level at different fermentation stages. (**C**,**D**) Random forest analysis identifying the main bacterial (**C**) and fungal (**D**) genera that discriminate between groups T and S. In the (**C**,**D**) panel, the heatmaps on the left show the relative abundance distribution of key differentially abundant genera across samples, with red and blue indicating high and low abundances, respectively. The bar plots on the right show the variable importance of each genus in the classification model.

At the bacterial phylum level, Bacteroidota and Proteobacteria together accounted for more than 80% of the communities in both groups at 0 d (Figure S2A). Firmicutes increased markedly after 10 d in both treatments. However, during the mid-to-late fermentation stages, Actinobacteriota progressively increased in group T and became predominant, whereas Firmicutes remained dominant in group S. At the genus level (Figure 2A), JC017 was highly abundant in both groups at 0 d. Clear divergence emerged after 10 d; group T became enriched in *Kroppenstedtia* and *Oceanobacillus* and subsequently developed a community dominated by Saccharopolyspora, whereas group S contained higher abundances of lactic acid bacteria, particularly *Weissella* and *Pediococcus*. By 40 d, Saccharopolyspora predominated in group T, whereas multiple genera, including *Kroppenstedtia* and *Weissella*, remained abundant in group S. These contrasting trajectories suggest that the initial wheat-associated microbiota contributed to the establishment of bacterial characteristics of high-temperature Daqu. These contrasting trajectories indicate that the initial wheat-associated microbiota influenced subsequent bacterial succession. The enrichment of thermotolerant taxa in group T suggests that early microbial differences may have favored the development of a community typical of high-temperature Daqu [21]. In contrast, reducing the wheat-associated microbiota altered the succession pattern in group S, which remained dominated by Firmicutes and lactic acid bacteria rather than shifting toward an Actinobacteriota-rich community [22].

Regarding the fungal communities, Ascomycota dominated the fungal communities throughout fermentation (>93%), with Basidiomycota and Mucoromycota present at relatively low abundances (Figure S2B). At the genus level (Figure 2B), group T was initially enriched in *Microascus* and *Thermoascus*, whereas group S contained higher abundances of *Alternaria* and *Rasamsonia*. At 20 d, Thermoascus increased sharply to 92.31% in group T. By 40 d, *Thermoascus* and *Thermomyces* co-dominated the community. In contrast, group S retained higher relative abundances of *Aspergillus*, *Saccharomycopsis*, and other fungi during the mid-to-late stages. The strong enrichment of *Thermoascus* in group T is consistent with more stringent thermal selection, whereas the broader fungal composition of group S suggests weaker or different selective pressure.

Random forest analysis further identified microbial taxa that contributed to the discrimination between the two Daqu groups. For bacterial communities (Figure 2C), *Saccharopolyspora*, *Actinopolyspora*, and *Caldibacillus* were characteristic of group T. In particular, the relative abundance of Saccharopolyspora reached 36.21% and 53.52% at 20 and 40 d, respectively, but remained below 9% throughout fermentation in group S. In contrast, *Weissella*, *Staphylococcus*, and *Pediococcus* were more strongly associated with group S. *Weissella* and *Pediococcus* were nearly undetectable in group T but remained abundant in group S from 10 to 40 d.

These differences were consistent with the distinct bacterial succession patterns observed between the two treatments. *Saccharopolyspora* and *Actinopolyspora* are thermotolerant actinobacteria commonly associated with high-temperature Daqu and are known for their capacity to degrade starch and proteins [19]. Their enrichment in group T therefore suggests stronger selection for thermotolerant bacterial taxa. In contrast, the persistent abundance of lactic acid bacteria in group S indicates that reducing the initial wheat-associated microbiota altered the subsequent bacterial succession trajectory and favored a community with different ecological characteristics [23].

For fungal communities (Figure 2D), *Thermoascus* was the main discriminatory genus associated with group T. Its relative abundance increased sharply to 92.31% at 20 d, making it the predominant fungal genus at this stage. In contrast, group S was characterized by a broader set of fungal taxa. *Rasamsonia* reached 19.67% at 20 d, while *Saccharomycopsis* was mainly detected in group S. At 40 d, *Thermomyces* and *Aspergillus* were also more abundant in group S than in group T. The enrichment of *Thermoascus* in group T was consistent with the reports of Zhang et al. [6] and Steindorff et al. [24], whereas the dominant bacterial genera differed between the two groups, suggesting that the subsequent succession of wheat-associated microbiota may depend on specific raw materials and Daqu-making conditions. In comparison, the persistence of *Rasamsonia*, *Saccharomycopsis*, *Thermomyces*, and *Aspergillus* in group S suggests a different fungal selection pattern under the altered fermentation conditions [25]. These differences further indicate that reduction in wheat-associated microorganisms affected not only bacterial succession but also the later composition of the fungal community. The persistence of molds and yeasts in group S may also contribute to the distinct volatile flavor profiles observed between the two treatments.

### 3.3. Microbial Niche Analysis

**Figure 3:**
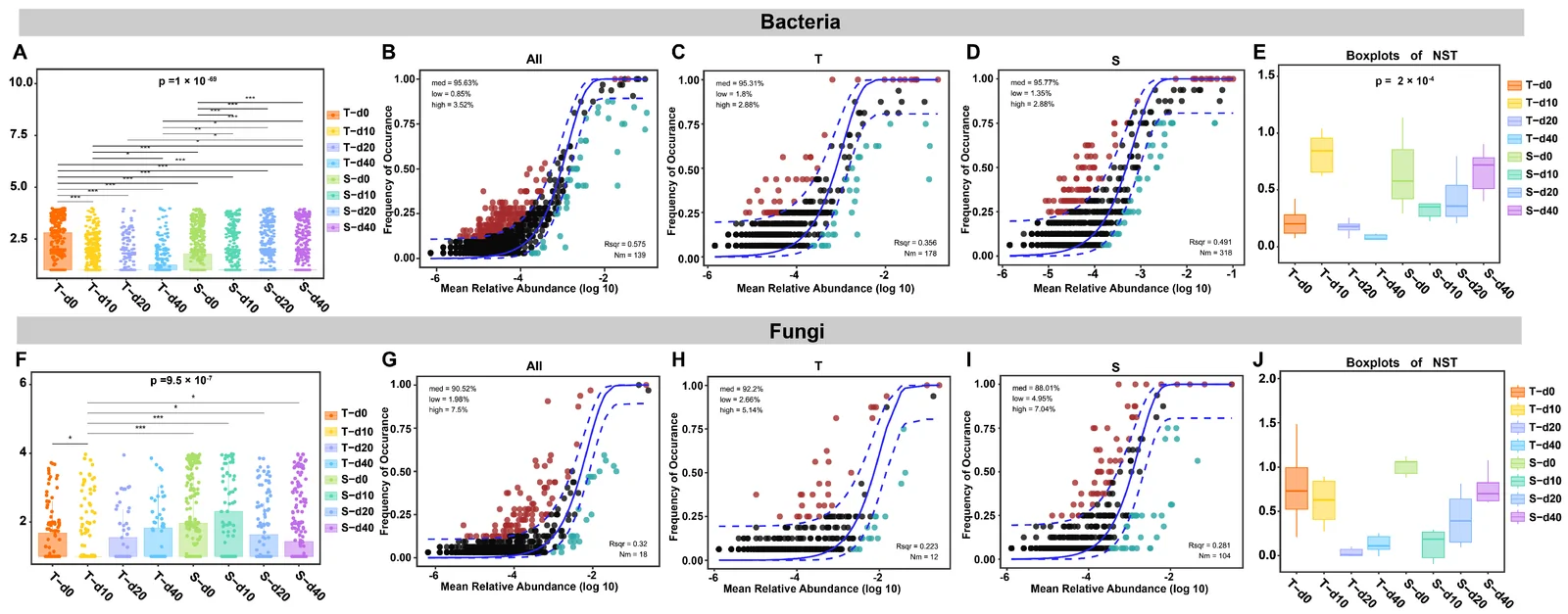
Ecological assembly processes and niche width of microbial communities during Daqu fermentation. (**A**,**F**) Comparison of niche width of bacterial (**A**) and fungal (**F**) communities at different fermentation stages. Asterisks indicate significant differences between groups (* *p* < 0.05, ** *p* < 0.01, and *** *p* < 0.001). (**B**–**D**,**G**–**I**) Sloan neutral community model (NCM) showing the relationship between the mean relative abundance of bacterial (**B**–**D**) and fungal (**G**–**I**) ASVs on a log10 scale and their occurrence frequency. Models were fitted to all samples (**B**,**G**), group T (**C**,**H**), and group S (**D**,**I**), respectively. The solid blue line represents the best-fit neutral model, and the blue dashed lines indicate the 95% confidence intervals. R^2^ represents the goodness of model fit, and Nm represents the product of community size and migration rate. In panels (**B**–**D**,**G**–**I**), colored dots represent microbial taxa grouped by their mean relative abundance: red indicates high abundance, black indicates medium/median abundance, and teal indicates low abundance. The proportions of each group are shown in the top-left corner of each panel. (**E**,**J**) Boxplots of the normalized stochasticity ratio (NST), showing the relative contributions of stochastic and deterministic processes to bacterial (**E**) and fungal (**J**) community assembly.

Niche width, the Sloan neutral community model, and the NST were used to characterize microbial community assembly (Figure 3). Bacterial niche width in group T remained relatively stable from 0 to 20 d but decreased markedly at 40 d, whereas group S showed stronger stage-specific fluctuations (Figure 3A), indicating distinct patterns of resource use and ecological adaptation. The Sloan neutral model showed moderate explanatory power for bacterial communities (Figure 3B–D), with R^2^ values of 0.575, 0.356, and 0.491 for the overall community, group T, and group S, respectively, and corresponding Nm values of 139, 178, and 318. NST values in group T exceeded 0.5 only at 10 d and were below 0.5 at 0, 20, and 40 d, whereas group S showed values above 0.5 at 0 and 40 d but below 0.5 at 10 and 20 d (Figure 3E). These results indicate stage-dependent bacterial assembly, with deterministic processes becoming more prominent in group T during the mid-to-late stages, while group S showed alternating contributions of stochastic and deterministic processes.

Fungal niche width decreased markedly during the mid-to-late stages in both groups (Figure 3F). The Sloan neutral model showed relatively low explanatory power for fungal communities, with R^2^ values of 0.320, 0.223, and 0.281 for the overall community, group T, and group S, respectively, and corresponding Nm values of 18, 12, and 104. The relatively low model fit suggests that neutral processes alone were insufficient to explain fungal succession [24]. Consistently, NST values in group T decreased sharply at 20 and 40 d and approached zero, whereas values in group S were below 0.5 at 10 and 20 d (Figure 3J). Together with the narrowing niche width, these patterns indicate a stronger contribution of deterministic processes to fungal community assembly during the mid-to-late stages. This deterministic signal was particularly pronounced in group T and coincided with the enrichment of thermotolerant fungi such as *Thermoascus* [25]. This stronger deterministic signal in group T during the mid-to-late stages was generally consistent with the environmental filtering effect reported for Daqu by Ma et al. [5]. However, the present analysis cannot distinguish the specific contributions of temperature, moisture, and acidity. In future practical Daqu production, these process parameters could be monitored together with community succession to further evaluate the effects of turning and ventilation regulation on batch stability. Nevertheless, stronger deterministic assembly should not be directly equated with better Daqu quality.

### 3.4. Volatile Flavor Compounds

#### 3.4.1. Volatile Flavor Profiles and Potential Contributing Compounds

**Figure 4:**
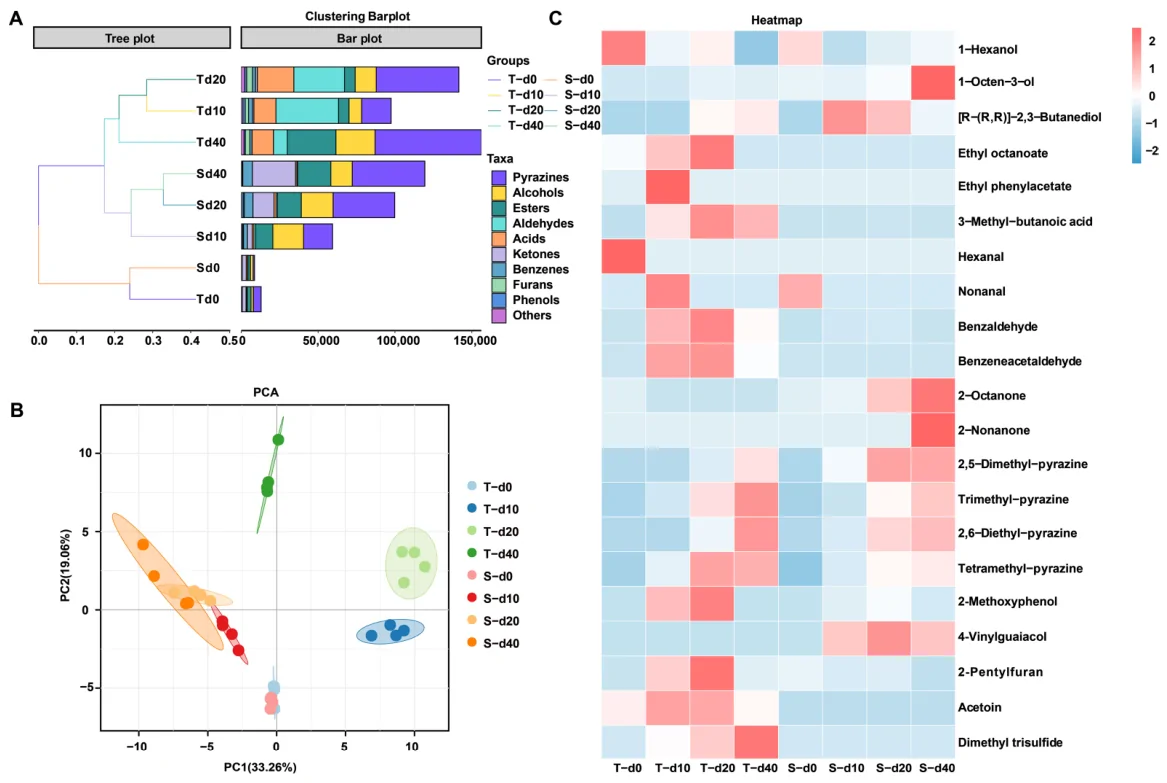
Comparative analysis of volatile flavor profiles during Daqu fermentation. (**A**) Hierarchical clustering dendrogram (**left**) and stacked bar plot showing the relative contents of major classes of volatile compounds (**right**). (**B**) Principal component analysis (PCA) score plot based on volatile compound profiles. (**C**) Dynamic changes in the relative abundances of potential aroma-contributing compounds with relative odor activity values (ROAV) > 1 in different Daqu samples. The color scale from blue to red represents standardized abundance values from low to high based on Z-scores.

A total of 110 volatile compounds were preliminarily identified, including 29 esters, 19 alcohols, 6 acids, 13 aldehydes, 9 ketones, 13 pyrazines, 4 phenols, 8 benzene compounds, 3 furans, and 6 other metabolites (Table S1). Composition analysis (Figure 4A) showed that different treatments significantly altered the accumulation patterns of volatile metabolites. In group T, pyrazines, esters, and acids were more prominent during the mid-to-late fermentation stages, and the relative contributions of acids and aldehydes changed between days 10 and 20. In contrast, the flavor succession in group S was relatively gradual and was consistently dominated by ketones, alcohols, and pyrazines at lower abundance, while the contents of acids and aldehydes remained at very low levels throughout the process. In principal component analysis, PC1 and PC2 explained 33.26% and 19.06% of the variance, respectively, and the two treatments exhibited distinct trajectories (Figure 4B). In group T, samples showed a marked shift in the principal component space from T0 to T40, with T10 and T20 mainly distributed on the positive side of PC1, whereas T40 shifted toward the positive direction of PC2. In contrast, samples from group S gradually shifted toward the negative side of PC1 during fermentation. At 40 d, T40 and S40 were clearly separated, indicating significant differences in the final volatile flavor profiles between the two groups.

By applying a threshold of ROAV ≥ 1, a total of 21 potential aroma-contributing compounds were identified (Figure 4C and Table S2), further revealing the essential differences between the two types of Daqu in characteristic aroma dimensions. In group T, the turning period (10–20 d) was characterized by significant enrichment of aldehydes, phenols, and sulfur-containing compounds, which had high aroma contributions. For example, benzaldehyde and phenylacetaldehyde, which impart caramel-like and sweet floral aromas, had ROAVs as high as 90.35 and 39.03 at 10 d, respectively; 2-methoxyphenol (guaiacol) and 4-vinylguaiacol, which contribute smoky and clove-like odors, and dimethyl trisulfide, which presents sulfurous and roasted aromas, showed extremely high aroma contributions in the mid-to-late period of group T. These compounds collectively constitute the typical roasted, sauce-like, and sweet floral composite flavor profile of traditional high-temperature Daqu [8]. In contrast, the key aroma compounds in group S exhibited a completely different aroma dimension. Among them, 1-octen-3-ol has a typical mushroom and earthy note and maintained a high ROAV throughout the whole stage of group S (up to 100); together with 2-nonanone, which contributes fruity and floral aromas, it conferred obvious fungal-like and delicate aroma characteristics to group S. The differences in aroma compounds strongly reflect the differences in substrate metabolic pathways between the two microecosystems. The enrichment of phenolic compounds and pyrazines in group T was consistent with the lignin and ferulic acid-degrading capacity of thermophilic actinomycetes and thermophilic fungi and with the typical high-temperature microenvironment. It also agreed with the report by Luo et al. [26] that temperature-related microbiota promote tetramethylpyrazine formation, suggesting that precursor supply and the fermentation environment may jointly drive flavor formation. In contrast, 1-octen-3-ol is a typical fungal secondary metabolite; group S retained a more diverse mold and yeast community, including *Aspergillus*, which may have promoted the continuous accumulation of fungus-related flavor compounds in group S. This indicates that the absence of wheat-derived microorganisms not only simplified the flavor profile but also fundamentally altered the aroma compound characteristics in Daqu [14,15].

#### 3.4.2. Screening of Differential Flavor Compounds

**Figure 5:**
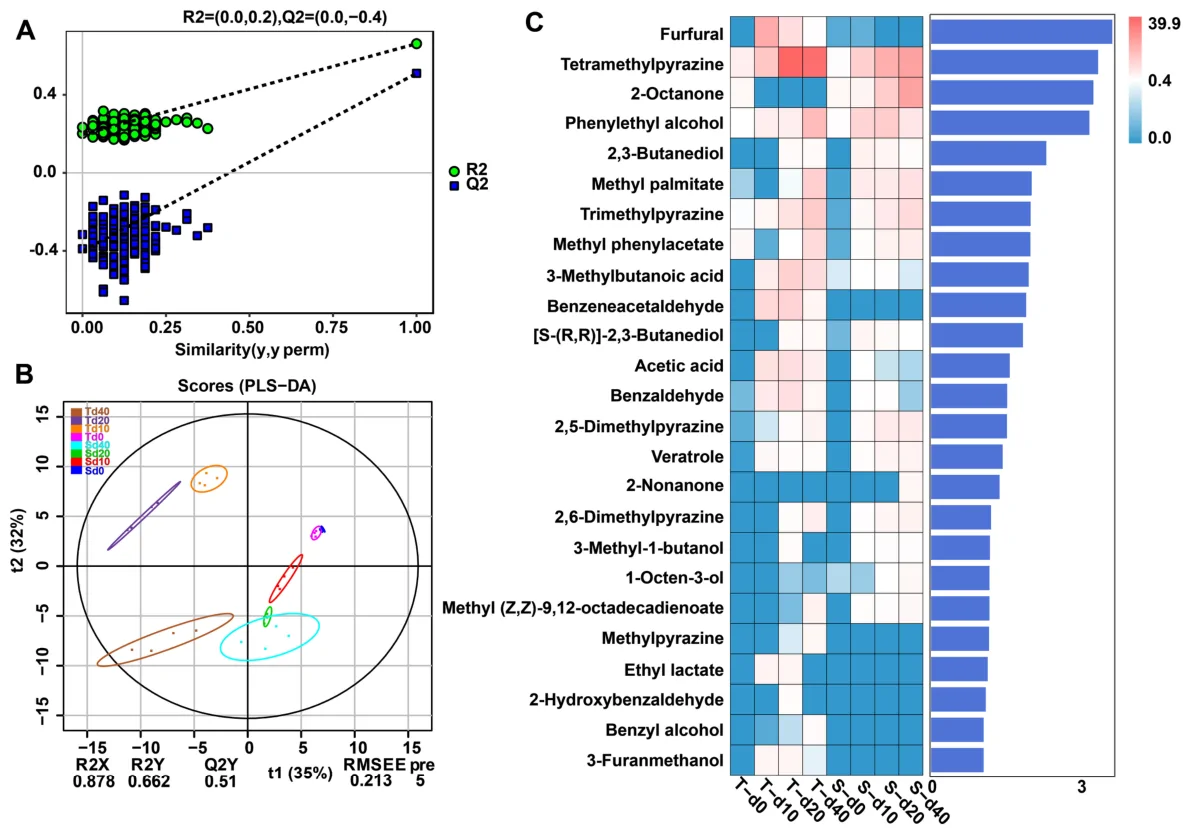
Identification of differential volatile flavor compounds during fermentation of traditional (T) and surface-disinfected (S) Daqu. (**A**) Permutation test of the partial least squares discriminant analysis (PLS-DA) model. (**B**) PLS-DA score plot. (**C**) Heatmap of significantly differential volatile compounds (**left**) and bar plot of their variable importance in projection (VIP) scores (**right**). Differential compounds were screened using the criteria of VIP > 1 and *p* < 0.05. In the heatmap, the color gradient from blue to red represents standardized relative abundance from low to high. The horizontal bars on the right indicate the corresponding VIP values.

To further identify the key compounds that distinguish the volatile profiles of the two types of Daqu, a PLS-DA model was established (Figure 5). The model exhibited acceptable explanatory and predictive performance (R^2^X(cum) = 0.878, R^2^Y(cum) = 0.662, Q^2^(cum) = 0.51), and its statistical robustness was validated by 200 permutation tests with no overfitting observed (Figure 5A). The score plot showed significant separation between group T and group S in the multidimensional space (Figure 5B). Based on this model, a total of 25 discriminant volatile markers (VIP > 1, *p* < 0.05) that contributed significantly to sample separation were screened (Figure 5C and Table S3). According to their dynamic concentration distribution patterns, these 25 differential markers could be clustered into three typical categories. The first category comprised compounds continuously enriched in group T, mainly including several pyrazines, such as tetramethylpyrazine and trimethylpyrazine, as well as 3-methylbutanoic acid and phenylacetaldehyde. These compounds accumulated substantially during the mid-to-late fermentation stages in group T, with tetramethylpyrazine reaching its highest level at 40 d, whereas their levels remained low or were undetectable in group S. The second category comprised stage-responsive compounds in group T, which exhibited transient peaks mainly during the turning-over period (10–20 d) of group T. For example, furfural accumulated sharply at 10 d in group T and then rapidly degraded, and ethyl lactate was also specifically enriched at this stage, while these compounds were almost undetectable in group S. The third category comprised compounds specifically enriched in group S, mainly including ketones such as 2-octanone, as well as higher alcohols such as phenylethanol and 1-octen-3-ol. These compounds increased continuously with the fermentation process in group S and maintained relatively high abundances during the mid-to-late stages, while their contents were extremely low or completely absent in group T.

The distribution of differential flavor markers reflected distinct fermentation microenvironments and metabolic pathways between the two groups. Pyrazines enriched in group T and furans showing stage-specific accumulation, particularly furfural, are typical products associated with high-temperature Maillard reactions [27]. Their accumulation was consistent with the stronger high-temperature fermentation characteristics of group T, which may have favored non-enzymatic browning reactions [26,28]. In addition, isovaleric acid and phenylacetaldehyde, which were preferentially enriched in group T, can arise from the catabolism of branched-chain and aromatic amino acids, respectively [12], and were associated with the distinct bacterial community composition of group T. In contrast, the markers preferentially enriched in group S were mainly associated with fungal and yeast metabolism. For instance, 1-octen-3-ol is commonly produced by fungi through lipoxygenase-mediated oxidation of linoleic acid [29], whereas phenylethanol is a typical product of yeast phenylalanine metabolism [30]. The sustained accumulation of these compounds in group S was consistent with the greater persistence of molds and yeasts under the relatively mild fermentation conditions observed in this group. Overall, reducing wheat-associated microorganisms altered microbial community succession and was accompanied by marked shifts in the accumulation of characteristic flavor compounds in Daqu.

### 3.5. Interaction Patterns of Microbial Communities

**Figure 6:**
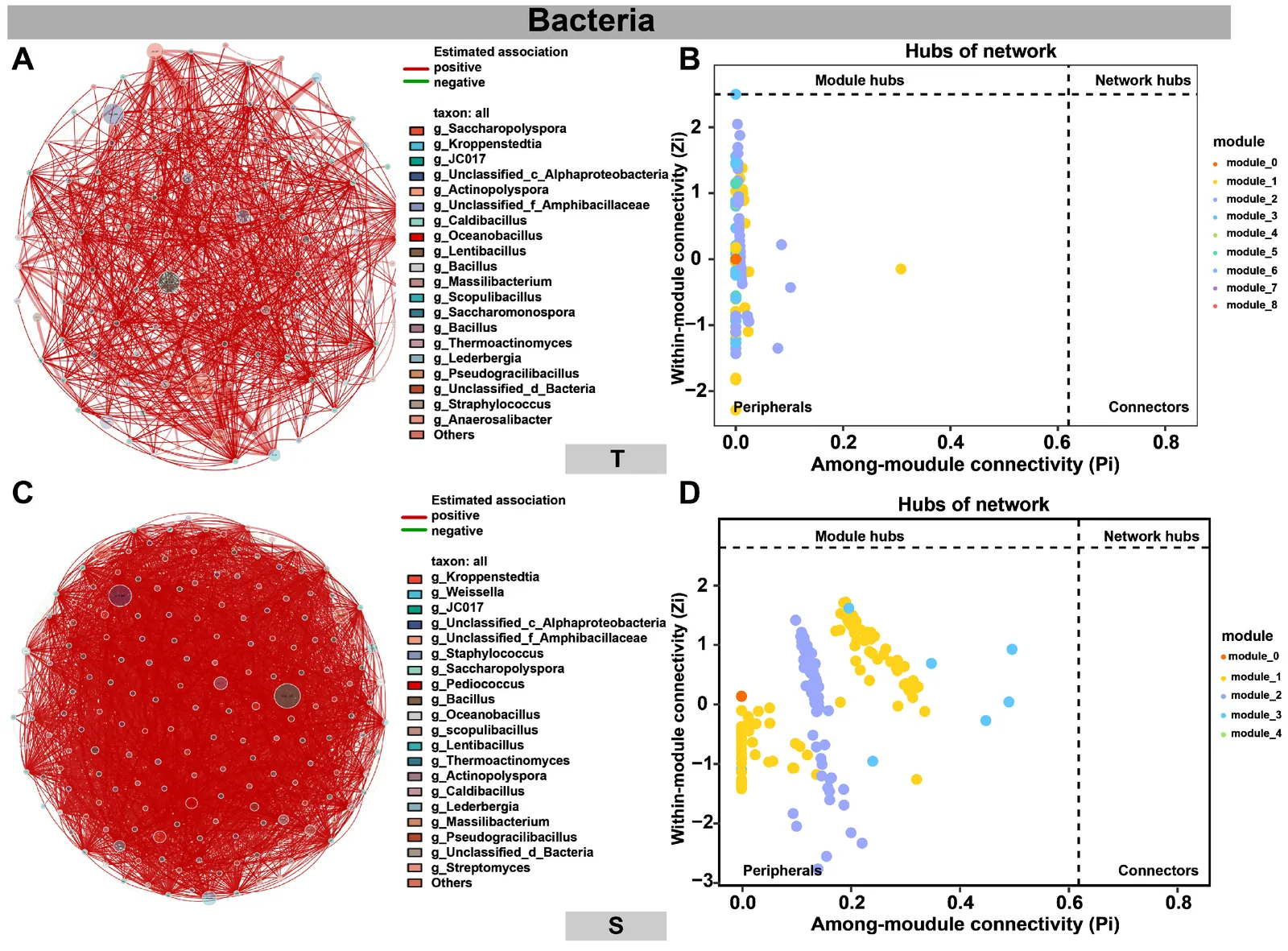

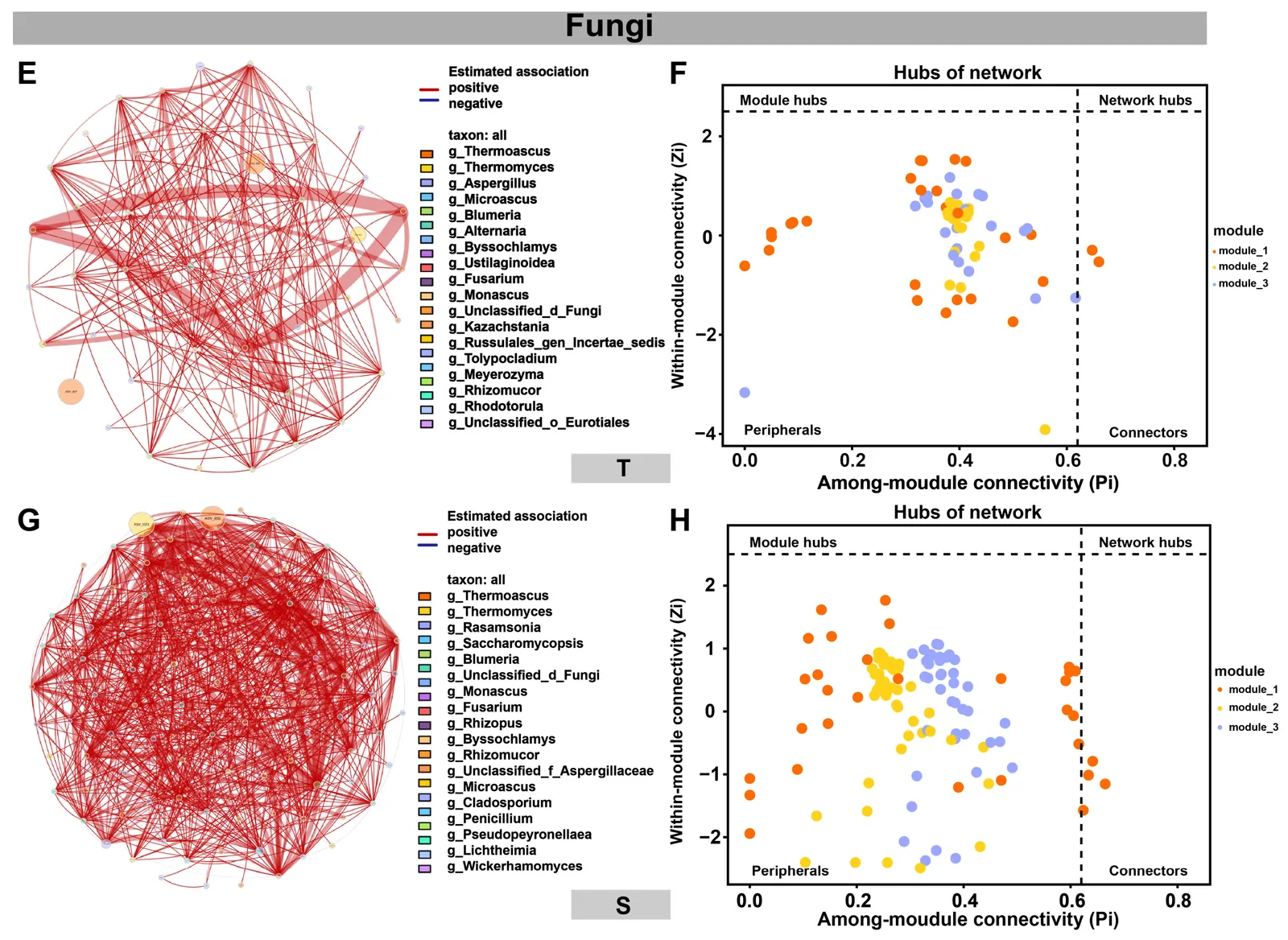
Co-occurrence networks and node topological roles of bacterial and fungal communities in groups T and S. (**A**,**C**) Bacterial and fungal networks of group T, respectively. (**E**,**G**) Bacterial and fungal networks of group S, respectively. Networks were constructed based on significant Spearman correlations. Nodes represent microbial taxa, node colors indicate taxonomic affiliation, and node size is proportional to node degree. Red edges indicate positive correlations, whereas green or blue edges indicate negative correlations. (**B**,**D**,**F**,**H**) Zi–Pi plots corresponding to each network, used to classify nodes as peripherals, connectors, module hubs, or network hubs.

Bacterial co-occurrence network analysis showed clear differences between groups T and S. The bacterial network of group T had relatively high modularity with a modularity index of 0.45 and contained 81 components (Figure 6A and Table S4). In contrast, group S showed a more connected network, with a clustering coefficient of 0.82 and a natural connectivity of 0.17. Its modularity index was lower at 0.33, and the network contained 20 components (Figure 6E and Table S4). Subnetwork analysis further showed different temporal patterns between the two groups (Table S5). In group T, the bacterial network was highly fragmented at 0 d, with an average of 52.5 components. The number of components decreased to 15.75 at 40 d, while network density gradually increased. This indicated increasing network connectivity during fermentation. In contrast, group S maintained a highly connected network from the beginning of fermentation, with about three components throughout the fermentation period. Zi-Pi analysis showed that most bacterial nodes in group T were peripheral nodes, whereas group S contained more module hubs (Figure 6B,F). These results indicate different patterns of bacterial community organization between the two treatments. Group T became more integrated while maintaining higher modularity, whereas group S remained more densely connected. These differences may be related to the initial microbial composition and subsequent environmental selection [31,32].

Fungal networks also showed different patterns between the two groups. The proportion of positive edges in the fungal networks was generally lower than that in the bacterial networks (Table S4). The fungal network of group T became simpler during the later stages and was mainly characterized by positive associations (Figure 6C). In contrast, group S retained more fungal taxa and a higher proportion of negative associations (Figure 6G). Zi-Pi analysis showed a more dispersed distribution of fungal nodes in group T, whereas several module hubs were present in group S (Figure 6D,H). Subnetwork analysis showed that the proportion of positive edges in group T increased markedly during fermentation. It reached 93.36% between 10 and 20 d and remained high thereafter. In group S, the proportion of positive edges ranged from 65.17% to 84.38% throughout fermentation (Table S5). The increase in positive associations in group T coincided with the simplification of the fungal community and the enrichment of thermotolerant fungi. This pattern may reflect similar responses of these taxa to the fermentation environment rather than direct cooperation [33].

### 3.6. Functional Prediction and Correlation Analysis

Based on PICRUSt2 functional prediction, both groups were enriched in functions related to carbohydrate metabolism (Figure S3). Among these, predicted functions associated with starch and polysaccharide degradation included α-amylase (EC 3.2.1.1) and glucan 1,4-α-glucosidase (EC 3.2.1.3). Sample-level temporal slope analysis showed that the mean temporal slopes of the α-amylase-associated function were negative in both groups T and S, whereas those of the glucan 1,4-α-glucosidase-associated function were positive in both groups (Table S6). These results reflect the overall direction of change over the study period; because the relevant changes deviated from linear trajectories, they cannot be used to infer that adjacent stages all changed in the same direction [12,34]. Both groups also harbored some predicted functions related to glycolysis, indicating partial overlap in their predicted functional composition.

The predicted results for both groups also included functions potentially related to flavor precursor metabolism. The alcohol dehydrogenase (EC 1.1.1.1) associated function showed an overall increasing trend in group T, whereas no significant linear temporal trend was detected in group S. The mean temporal slope of the acetolactate synthase (EC 2.2.1.6) associated function was positive in group T, but did not reach statistical significance in group S (Table S6). The predicted results also included functions related to 2,3-butanediol dehydrogenase. Carboxylesterase (EC 3.1.1.1) and triacylglycerol lipase (EC 3.1.1.3), which are related to lipid and ester metabolism [31], had significantly positive mean temporal slopes in both groups, but their changes deviated from linear trajectories and should therefore be interpreted as overall directions of change (Table S6).

In addition, the predicted results included functions potentially involved in the transformation of aromatic compounds, namely aromatic L-amino acid decarboxylase (EC 4.1.1.28) and monoamine oxidase (EC 1.4.3.4). The mean temporal slopes of both functions were significantly positive in groups T and S, but both exhibited non-linear changes (Table S6). The catechol O-methyltransferase (EC 2.1.1.6)-associated function was excluded because it did not meet the requirement for the proportion of non-zero samples. These predicted results provide clues for exploring the potential transformation of aromatic precursors, but they cannot be used to confirm the actual production of phenols, aromatic alcohols, or other flavor compounds [35,36]. Notably, the above analysis represents an exploratory assessment of functional potential and should not be interpreted as a direct measurement of functional gene abundance, enzyme expression, or enzyme activity.

**Figure 7:**
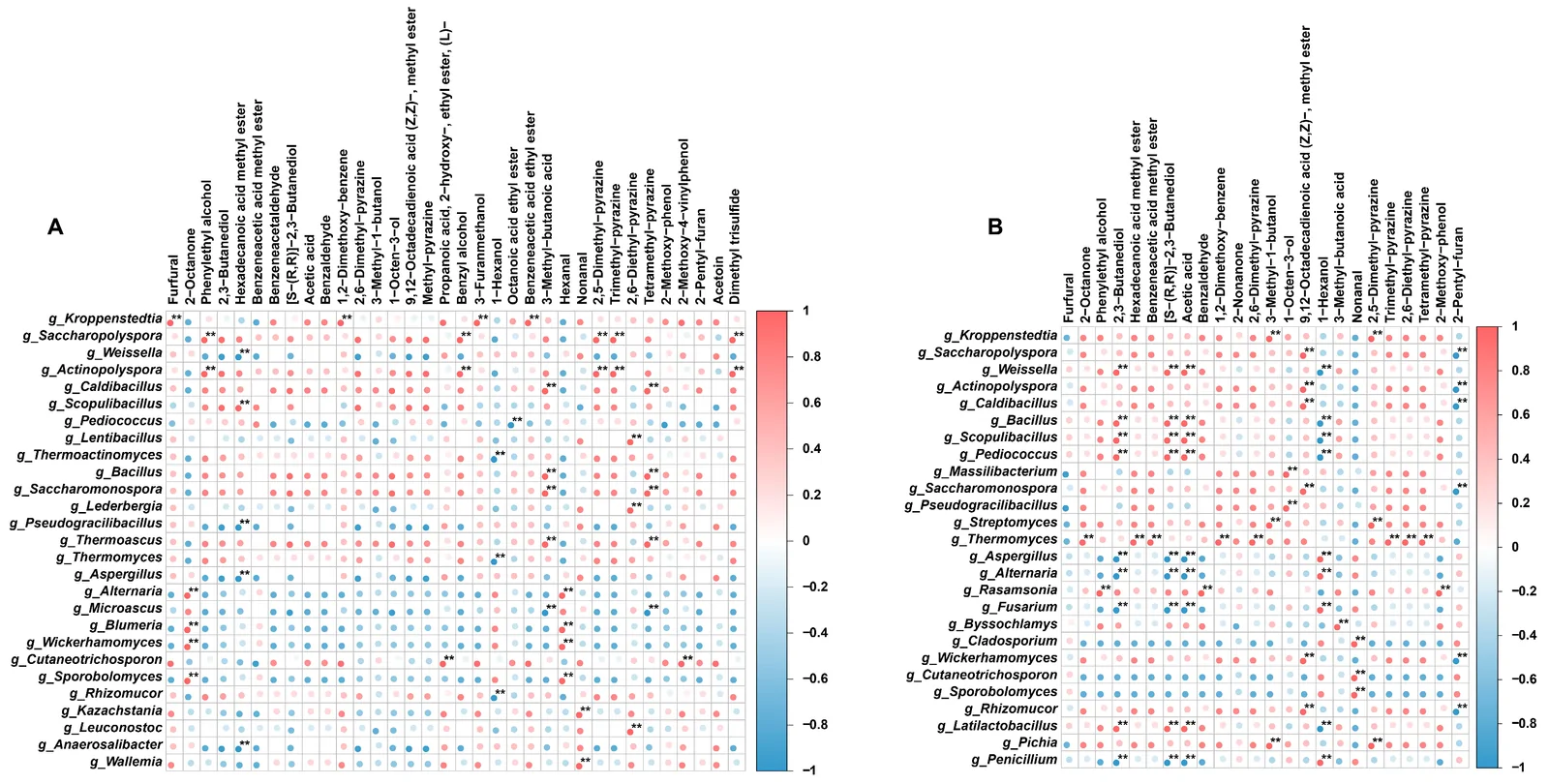
Correlations between key and dominant microorganisms and differential or characteristic volatile compounds in the (**A**) T and (**B**) S groups. Heatmaps were constructed based on Pearson correlation coefficients, showing the relationships between selected key and dominant microorganisms and differential or characteristic volatile compounds. Dot color indicates the direction of correlation, with red and blue representing positive and negative correlations, respectively. Color intensity and dot size indicate correlation strength. In the figure, asterisks (**) indicate a highly significant difference (*p* < 0.01).

Pearson correlation analysis was further used to explore associations between dominant microorganisms and characteristic volatile compounds (Figure 7A,B). In group T, *Saccharopolyspora* and *Actinopolyspora* were positively correlated with dimethyl trisulfide and several pyrazines, including 2,5-dimethylpyrazine and trimethylpyrazine. These associations suggest that the enrichment of thermotolerant actinobacteria coincided with the accumulation of sulfur-containing compounds and pyrazines during traditional Daqu fermentation [37]. Among fungal taxa, *Cutaneotrichosporon* was positively correlated with 2-methoxy-4-vinylphenol, while *Bacillus* showed a strong positive correlation with 3-methylbutyric acid. These relationships are consistent with reported links between microbial metabolism, ferulic acid conversion, and amino acid catabolism [12], but do not establish direct biosynthetic contributions.

In group S, *Weissella*, *Pediococcus*, and *Bacillus* were positively associated with acetic acid and 2,3-butanediol, suggesting a potential relationship with pyruvate-derived metabolism. Thermomyces was positively associated with several esters and heterocyclic compounds, including methyl phenylacetate and tetramethylpyrazine [38]. Its known macromolecule-degrading capacity may increase the availability of amino acids and reducing sugars, which could indirectly influence downstream flavor-forming reactions. Overall, functional prediction indicated that the two groups shared substantial potential for primary carbohydrate metabolism, whereas their associations with characteristic secondary metabolites differed more clearly. These differences were consistent with the distinct microbial succession patterns and volatile profiles observed between groups T and S.

## 4. Conclusions

This study evaluated the contribution of wheat-associated microorganisms to microbial succession, community assembly, and volatile flavor formation during high-temperature Daqu fermentation. Traditional Daqu (T) developed communities characterized by *Saccharopolyspora* and *Thermoascus*, whereas the sterilized-wheat (S) group retained higher abundances of lactic acid bacteria and a broader range of molds and yeasts. Community assembly analyses indicated a stronger contribution of deterministic selection during the mid-to-late stages of traditional Daqu fermentation, particularly for fungi. The two treatments also developed distinct volatile profiles. Traditional Daqu was characterized by higher contributions of tetramethylpyrazine, dimethyl trisulfide, and 2-methoxy-4-vinylphenol, whereas 1-octen-3-ol and 2-octanone were more characteristic of the sterilized-wheat group. Although functional prediction suggested substantial redundancy in primary carbohydrate metabolism, microbial composition was differentially associated with characteristic secondary metabolites. Collectively, these results demonstrate that the initial wheat-associated microbiota contributes substantially to microbial succession and flavor development during high-temperature Daqu fermentation. These findings provide a basis for further investigation of raw-material microbiota and their potential application in improving the consistency and quality of Daqu production. In the future, key functional microorganisms could be isolated and screened, directed supplementation or fortified starters could be developed, and process regulation could be combined to construct a controllable initial community, thereby stabilizing the fermentation process and flavor and promoting standardized production applications. However, this study is only an exploratory correlation analysis and did not integrate the microbial community, PICRUSt2-predicted functions, and volatile profiles; the predicted functions are not independent measurements, and the conclusions still require validation by metatranscriptomics, metaproteomics, or targeted metabolomics.

## Data Availability

The original contributions presented in the study are included in the article/Supplementary Materials, and further inquiries can be directed to the corresponding authors.

## Supplementary Materials

The following supporting information can be downloaded at: https://www.mdpi.com/article/10.3390/foods15193511/s1, Table S1. Identification of Volatile Compounds in Different Daqu Samples (n = 3); Table S2. Aroma-Active Compounds Identified Based on ROAV Values; Table S3. Differential Compounds Identified by PLS-DA for Discriminating among Different Daqu Samples; Table S4. Topological Properties of Microbial Co-occurrence Networks; Table S5. Topological Properties of Microbial Subnetworks; Table S6. Statistical analysis of enzymes derived from bacteria and fungi; Figure S1. Alpha-diversity indices of bacterial communities at different fermentation stages (A–D). Alpha-diversity indices of fungal communities at different fermentation stages (E–H). (T represents traditional naturally fermented Daqu, S represents Daqu fermented with surface-disinfected wheat, and D0, D10, D20, and D40 represent fermentation days 0, 10, 20, and 40, respectively.); Figure S2. Microbial Community Composition at the Phylum Level during Daqu Fermentation; Figure S3. Abundance of Microbial Enzymes Related to Flavor Compound Formation during Daqu Fermentation. (A) Bacteria-derived enzymes; (B) fungi-derived enzymes. Refs [39,40,41,42,43,44,45,46,47] are cited in the Supplementary Materials.

## Abbreviations

The following abbreviations are used in this manuscript:

T	Traditionally fermented Daqu
S	Sterilized wheat Daqu
GC–MS	Gas chromatography–mass spectrometry
PCoA	Principal coordinate analysis
NST	Normalized stochasticity ratio
PLS-DA	Partial least squares discriminant analysis
KEGG	Kyoto Encyclopedia of Genes and Genomes
VIP	Variable importance in projection
ROAV	Relative odor activity values
