# Supplementary material for: Wheat-Surface Microbiota as a Key Driver of Daqu Quality: Evidence from Microbial Diversity and Volatile Flavor Analysis
Source: Foods. 2026 Oct 1;15(19):3511. doi: 10.3390/foods15193511 (PMC13664550; doi:10.3390/foods15193511)
Supplement: Supplementary file 1 [file foods-15-03511-s001.zip › foods-4550262-supplementary.pdf]

Supplemental Materials

Wheat-surface microbiota as a key driver of Daqu quality: Evidence from microbial diversity and volatile flavor analysis

Ying Huang[1], Huan Guo[2], Hao Liu[1], Fang Wei[1], Hong Deng[3], Hong Zhang[2], Li Jiang[4*], Yu, Mu[2],*

[1] School of Brewing Engineering, Moutai Institute, Renhuai 564507, China

[2] School of Food Engineering, Moutai Institute, Renhuai 564507, China

[3] School of Resources and Environment Engineering, Moutai Institute, Renhuai 564507, China

[4] School of Liquor and Food Engineering, Guizhou University, Guiyang, 550025, China

* Correspondence: jiangli0014@163.com; Tel.: +86 18985822153; mu_yu1997@163.com; Tel.: +86 18848469453

Table S1. Identification of Volatile Compounds in Different Daqu Samples (n = 3)

RT	Compounds	CAS	T0	T10	T20	T40	S0	S10	S20	S40
13.6833	1-Methoxy-2-propanol	107-98-2	0±0	0±0	0±0	0±0	0±0	0±0	86.97±10.59	249.65±76.66
16.3143	3-Methyl-1-butanol	123-51-3	0±0	0±0	1314.18±161.34	0±0	0±0	472.66±57.6	890.43±77.35	809.55±20.5
18.3565	1-Pentanol	71-41-0	0±0	0±0	0±0	0±0	172.42±20.91	0±0	0±0	0±0
20.6963	Prenol	556-82-1	0±0	0±0	0±0	0±0	0±0	44.1±4.14	0±0	0±0
21.3027	1-Hexanol	111-27-3	902.84±52.66	396.05±58.6	495.54±30.14	167.94±18.34	591.86±65.43	267.6±17.75	362.46±24.65	407.19±13.91
24.135	1-Octen-3-ol	3391-86-4	0±0	0±0	203.64±11.73	143.03±83.3	245.2±24.72	199.36±74.95	401.25±33.88	2428.53±121.18
24.3546	1-Heptanol	111-70-6	98.98±11.57	0±0	0±0	0±0	72.92±10.01	37.73±2.06	46.59±7	46.05±6.99
26.3283	2-Nonanol	628-99-9	0±0	0±0	0±0	0±0	0±0	0±0	0±0	391.06±23.37
26.5984	[S-(R,R)]-2,3-Butanediol	19132-06-0	0±0	0±0	1937.2±87.9	1936.01±150.56	115.07±15.86	2866.62±681.29	1731.9±56.17	513.76±98.06
26.9546	1-Octanol	111-87-5	19.06±7.68	0±0	35.17±2.31	0±0	36.02±3.34	25.11±7.91	32.15±4.89	43.78±10.11
27.4359	[R-(R*,R*)]-2,3-Butanediol	24347-58-8	0±0	0±0	1688.33±92.3	2032.69±279.73	0±0	4138.61±176.6	3032.58±135.6	1126.61±126.66
28.6387	(E)-2-Octen-1-ol	18409-17-1	0±0	0±0	0±0	0±0	0±0	0±0	0±0	0±0
29.4015	3-Furanmethanol	4412-91-3	0±0	3046.06±155.24	2902.67±64.13	350.32±33.83	0±0	0±0	0±0	0±0
29.5749	1-Nonanol	143-08-8	0±0	0±0	0±0	0±0	0±0	0±0	37.39±4.11	40.05±28.06
30.5984	3-(Methylthio)-1-propanol (Methionol)	505-10-2	0±0	0±0	127.84±12.81	0±0	0±0	37.88±11.89	0±0	0±0
33.7275	Benzyl alcohol	100-51-6	0±0	67.74±39.11	268.59±24.81	1940.21±142.24	0±0	0±0	0±0	0±0
34.7196	Phenylethyl alcohol	60-12-8	439.48±21.68	4679.38±174.86	4720.19±145.92	18274.52±301.9	546.39±43.99	11658.57±1358.99	13596.29±323.29	7189.26±940.54
34.9968	β-Methylphenethyl alcohol	1123-85-9	0±0	0±0	0±0	0±0	0±0	95.32±12.81	104.09±11.6	231.85±67.95
35.5921	β-Ethylphenethyl alcohol	2035-94-1	0±0	0±0	0±0	589.18±83.99	0±0	148.14±38.4	397.46±13.9	434.91±102.08
15.3166	Methyl hexanoate	106-70-7	75.26±14.27	0±0	0±0	1139.78±46.58	1041.7±134.01	804.14±39.27	1231.27±234.72	967.25±160.05

17.2432	Ethyl hexanoate	123-66-0	0±0	252.4±59.38	139.38±10.84	106.29±64.72	0±0	0±0	0±0	0±0
17.4564	Methyl 5-methylhexanoate	2177-83-5	0±0	0±0	0±0	376.64±29.31	0±0	0±0	0±0	0±0
18.6028	Hexyl acetate	142-92-7	0±0	76.46±44.22	42.67±7.2	0±0	0±0	0±0	0±0	0±0
20.595	Ethyl heptanoate	106-30-9	0±0	78.99±13.47	62.69±8.72	0±0	0±0	0±0	0±0	0±0
21.0481	Ethyl (L)-2-hydroxypropanoate	687-47-8	0±0	3389.04±187.27	2867.19±78.13	0±0	0±0	0±0	0±0	0±0
23.825	Ethyl octanoate	106-32-1	89.61±11.9	261.5±17.78	468.37±63.48	0±0	0±0	0±0	0±0	0±0
25.5684	Methyl nonanoate	1731-84-6	0±0	0±0	0±0	0±0	53.19±8.39	91.54±18.65	192.41±27.26	191.98±49.41
28.2078	Methyl decanoate	110-42-9	0±0	0±0	0±0	0±0	0±0	193.94±51.54	225.66±81.99	194.6±57.3
28.7772	Methyl benzoate	93-58-3	0±0	354.18±27.38	247.55±32.52	1227.26±214.25	362.27±19	483.48±29.82	786.37±41.11	893.14±139.61
28.7806	Methyl 4-decenoate	1191-02-2	0±0	0±0	0±0	0±0	0±0	43.29±17.65	87.52±12.97	101.5±31.09
29.6802	Ethyl benzoate	93-89-0	0±0	194.11±11.15	0±0	0±0	0±0	0±0	0±0	0±0
29.7709	Ethyl trans-4-decenoate	76649-16-6	0±0	0±0	0±0	0±0	0±0	108.78±17.29	53.72±6.23	78.19±12.85
31.884	Methyl phenylacetate	101-41-7	2186.23±45.14	76.64±6.73	493.47±42.51	9591.37±379.13	81.09±9.73	1044.36±131.42	3714.92±83.46	5410.01±250.92
32.0185	Ethyl phenylacetate	101-97-3	0±0	1785.4±380.88	1662.59±155.43	304.4±79.44	0±0	0±0	0±0	0±0
32.4929	Methyl dodecanoate	111-82-0	0±0	0±0	0±0	0±0	0±0	127.73±25.45	105.18±12.76	0±0
33.1832	Methyl phenylpropanoate	103-25-3	0±0	0±0	0±0	86.56±6.73	0±0	0±0	0±0	0±0
33.9013	Ethyl phenylpropanoate	2021-28-5	0±0	66.9±4.5	52.82±3.71	0±0	0±0	0±0	0±0	0±0
35.8934	Methyl tetradecanoate	124-10-7	0±0	0±0	0±0	390.65±36.02	0±0	554.04±117.01	698.36±23.26	945.14±276.52
37.0562	Methyl 13-methyltetradecanoate	1000424-50-7	0±0	0±0	0±0	259.37±84.68	0±0	69.82±11.67	400.05±10.55	730.69±100.99
37.6033	Methyl pentadecanoate	7132-64-1	0±0	0±0	0±0	185.7±56.89	0±0	369.88±96.6	452.2±22.92	747.39±207.99
39.2412	Methyl palmitate	112-39-0	217.73±25.53	0±0	375.27±84.58	12722.96±225.92	39.77±3.27	5446.46±152.7	5817.25±169.42	8167.81±326.82
39.7983	Ethyl palmitate	628-97-7	0±0	200.39±18.54	276.33±40.13	486.98±72.41	0±0	0±0	0±0	0±0
42.6164	Methyl stearate	112-61-8	0±0	0±0	0±0	0±0	0±0	91.46±34.02	138.35±35.42	171.24±30.98

42.6479	Methyl (E)-9-octadecenoate	1937-62-8	0±0	0±0	0±0	832.27±32.41	0±0	0±0	0±0	0±0
42.9918	Methyl (Z)-9-octadecenoate	112-62-9	0±0	0±0	0±0	0±0	0±0	418.34±40.15	601.52±33.3	889.72±50
43.4237	Methyl (Z,Z)-9,12-octadecadienoate	112-63-0	0±0	0±0	140.99±25.85	3957.4±144.69	0±0	1232.57±92.5	902.2±150.79	2108.52±490.61
43.9888	Ethyl linoleate	544-35-4	0±0	61.91±18.77	105.35±12.06	0±0	0±0	0±0	0±0	0±0
45.0252	Methyl linolenate	301-00-8	0±0	0±0	0±0	0±0	0±0	51.88±6.27	47.54±4.87	66.11±9.16
24.2716	Acetic acid	64-19-7	0±0	7861.06±110.56	8800.6±201.74	4355.17±877.83	0±0	446.09±31.09	290.61±17.28	241.27±45.38
27.6155	2-Methylpropanoic acid	79-31-2	77.59±16.4	692.6±89.94	1856.14±184.16	729.73±59.57	15.17±1.86	86.78±5.85	316.96±69.28	229.06±90.07
29.4777	3-Methylbutanoic acid	503-74-2	0±0	5354.42±138.57	11987.72±129.74	8868.66±846.69	321±14.47	406.69±85.48	657.1±50.23	316.9±31.44
32.1655	4-Methylpentanoic acid	646-07-1	0±0	0±0	0±0	0±0	0±0	0±0	0±0	0±0
33.0017	Hexanoic acid	142-62-1	0±0	428.15±22.86	407.56±49.06	0±0	521.44±29.15	49.27±5.86	0±0	0±0
44.6815	Phenylacetic acid	103-82-2	0±0	0±0	684.37±88.69	0±0	0±0	75.1±6.34	101.87±16.2	161.32±18.73
11.7436	Hexanal	66-25-1	390.82±13.31	0±0	0±0	0±0	0±0	0±0	0±0	0±0
20.5688	(E)-2-Heptenal	18829-55-5	65.2±2.38	0±0	0±0	0±0	74.23±12.64	0±0	0±0	0±0
21.6272	2-Isopropyl-5-methyl-2-hexenal	35158-25-9	0±0	0±0	141.28±82.49	0±0	0±0	0±0	0±0	0±0
22.9802	Nonanal	124-19-6	0±0	105.27±12.29	0±0	0±0	79.08±11.29	0±0	0±0	0±0
24.9131	Furfural	98-01-1	0±0	22413.16±723.39	9295.56±497.06	580.52±37.89	64.28±10.45	88.45±8.12	0±0	0±0
26.4014	(E)-2-Nonenal	18829-56-6	0±0	14.53±4.55	0±0	0±0	0±0	0±0	0±0	0±0
26.5016	Benzaldehyde	100-52-7	133±30.87	6124.9±334.74	8441.46±62.06	2643.91±150.44	0±0	604.23±27.45	696.06±79.59	202.91±27.9
27.7521	5-Methyl-2-furfuraldehyde	620-02-0	0±0	549.61±54	445.69±16.82	150.67±55.39	0±0	0±0	0±0	0±0
29.2479	Phenylacetaldehyde	122-78-1	0±0	10581.37±185.92	11855.39±51.04	3110.1±194.35	0±0	0±0	0±0	0±0
30.1162	2-Hydroxybenzaldehyde	90-02-8	0±0	0±0	1494.4±84.97	0±0	0±0	0±0	0±0	0±0

32.4665	2-Phenylpropenal	4432-63-7	0±0	0±0	0±0	2178.71±798.62	0±0	510.38±40.85	229.06±40.67	233.71±72.23
34.8737	α-Ethylidenephenylacetaldehyde	4411-89-6	0±0	533.02±14.84	610.26±16.97	0±0	0±0	0±0	0±0	0±0
37.2229	5-Methyl-2-phenyl-2-hexenal	21834-92-4	0±0	399.52±75.46	677.56±63.1	253.3±84.03	0±0	0±0	0±0	0±0
18.3261	3-Octanone	106-68-3	0±0	0±0	0±0	0±0	0±0	0±0	0±0	541.77±71.08
18.992	2-Octanone	111-13-7	2349.13±99.81	0±0	0±0	0±0	2514.95±251.15	3197.15±179.33	13535.68±423.63	24408.53±6743.74
22.8797	2-Nonanone	821-55-6	0±0	0±0	0±0	0±0	0±0	0±0	0±0	2757.34±784.81
27.6903	1-(2-Furanyl)-1-propanone	3194-15-8	0±0	278.66±11.29	421.79±42.54	0±0	0±0	0±0	0±0	0±0
29.1518	4-(2-Furanyl)-2-butanone	699-17-2	0±0	76.73±7.04	161.82±11.57	0±0	0±0	0±0	0±0	0±0
29.7902	Acetophenone	98-86-2	0±0	0±0	0±0	0±0	0±0	0±0	140.37±83.36	280.39±85.12
30.9268	5-Ethyldihydro-2(3H)-furanone	695-06-7	0±0	0±0	0±0	0±0	58.73±6.5	0±0	0±0	0±0
32.5841	1-(2-Furanyl)-1-pentanone	3194-17-0	0±0	0±0	80.97±5.82	0±0	0±0	0±0	0±0	0±0
36.5921	Dihydro-5-pentyl-2(3H)-furanone	104-61-0	170.17±71.93	549.1±6.52	513.79±60.84	0±0	70.19±4.05	0±0	0±0	0±0
18.7238	Methylpyrazine	109-08-0	0±0	0±0	320.43±91.25	2379.36±164.43	0±0	0±0	0±0	0±0
20.6477	2,5-Dimethylpyrazine	123-32-0	83.06±12.03	309.64±52.88	1341.92±44.4	3495.73±679.29	0±0	2064.89±148.1	5888.95±215.9	5812.45±249.89
21.1022	2,6-Dimethylpyrazine	108-50-9	0±0	0±0	1262.44±83.6	4780.06±80.8	0±0	761.96±51.3	2926.21±61.35	3644.47±172.75
21.4402	2,3-Dimethylpyrazine	5910-89-4	138.98±10.72	308.77±38.96	1219.25±12.17	2480.81±122.84	0±0	251.95±75	896.38±14.3	942.39±178.81
22.5131	2-Ethyl-6-methylpyrazine	13925-03-6	0±0	0±0	0±0	666.04±67.87	0±0	185.81±17.22	793.06±41.88	921.78±221.76
22.6926	2-Ethyl-5-methylpyrazine	13360-64-0	0±0	0±0	0±0	365.65±83.35	0±0	0±0	0±0	0±0
23.1615	Trimethylpyrazine	14667-55-1	388.3±23.87	2693.42±667.87	8009.96±322.55	13760.36±138.31	74.08±4.71	2212.8±599.93	6074.92±773.59	9657.87±981.62
24.7034	2,3-Dimethyl-5-ethylpyrazine	15707-34-3	0±0	0±0	1036.18±182.73	1581.58±72.42	0±0	0±0	0±0	0±0
25.0173	2,6-Diethylpyrazine	13067-27-1	218.28±33.72	464.96±35.7	123.6±12.42	247.26±43.44	23.41±1.94	154.35±11.22	1118.11±79.93	1226.9±572.01

25.0956	Tetramethylpyrazine	1124-11-4	4270.2±12.11	15148.92±524.96	39894.09±353.1	37465.48±490.53	804.6±83.56	12790.53±194.18	21262.45±109.42	24378.18±523.96
25.5635	2-Vinyl-6-methylpyrazine	13925-09-2	0±0	0±0	0±0	1165.02±100.76	0±0	370.32±64.15	651.49±40.77	199.76±26.23
26.0413	3,5-Diethyl-2-methylpyrazine	18138-05-1	0±0	241.57±17.39	583.52±16.26	734.14±33.36	0±0	0±0	0±0	0±0
26.3404	2,3,5-Trimethyl-6-ethylpyrazine	17398-16-2	0±0	0±0	0±0	0±0	0±0	76.03±15.26	487.19±27.75	413.61±53.63
33.8437	2-Methoxyphenol	90-05-1	0±0	335.04±70.97	504.15±12.11	0±0	0±0	63.82±12.89	115.07±27.17	36.91±2.87
35.9823	Phenol	108-95-2	18.16±2.04	360.99±74.93	410.83±27.79	888.25±160.39	0±0	183.97±65.7	280.8±71.29	0±0
36.7798	4-Ethylguaiacol	2785-89-9	0±0	0±0	0±0	0±0	0±0	505.36±87.18	792.77±30.2	532.36±27
39.1799	4-Vinylguaiacol	7786-61-0	0±0	859.29±26.49	300.5±7.14	0±0	0±0	0±0	0±0	0±0
29.9769	p-Methoxystyrene	637-69-4	0±0	72.02±1.64	0±0	0±0	0±0	0±0	0±0	0±0
30.8508	Veratrole	91-16-7	20.74±2.33	2168.87±25.33	1499.17±84.95	1072.48±84.46	0±0	1079.08±213.7	3243.72±45.93	3491.33±983.23
31.1816	p-Dimethoxybenzene	150-78-7	0±0	0±0	0±0	191.34±12.11	0±0	81.09±4.54	231.73±85.61	285.52±21.65
34.0799	4-Ethyl-1,2-dimethoxybenzene	5888-51-7	0±0	0±0	0±0	0±0	0±0	792.88±139.26	613.64±12.96	429.27±31.38
34.822	1-Isocyano-3-methylbenzene	20600-54-8	0±0	195.37±13	225.47±30.42	0±0	0±0	0±0	0±0	0±0
35.235	1,2,3-Trimethoxybenzene	634-36-6	0±0	144.96±13.5	273.49±42.34	184.18±25.89	0±0	51.67±5.72	101.44±25.71	177.73±81.94
36.838	4-Vinyl-1,2-dimethoxybenzene	6380-23-0	0±0	0±0	0±0	0±0	0±0	306.4±55.54	960.46±29.08	1244.31±476.47
37.6728	1,2,4-Trimethoxybenzene	135-77-3	0±0	0±0	0±0	0±0	0±0	134.05±48.54	424.27±40.37	669.25±24.26
25.9421	Benzofuran	271-89-6	0±0	57.18±3.01	0±0	0±0	0±0	0±0	0±0	0±0
17.1105	2-Pentylfuran	3777-69-3	298.84±10.66	1534.81±6.7	2802.38±172.66	553.41±37.46	647.58±76.15	453.46±88.99	500.25±57.89	205.29±28.01
33.4424	3-Phenylfuran	13679-41-9	0±0	562.49±13.17	961.41±68.27	2380.69±69.2	0±0	84.32±20.99	197.19±52.95	205.27±34.02
16.2155	3-(1-Methylethyl)oxetane	10317-17-6	0±0	0±0	830.02±81.07	0±0	0±0	0±0	0±0	0±0

17.9988	3-Carene	13466-78-9	0±0	0±0	0±0	0±0	0±0	0±0	164.1±65.45	0±0
19.1209	Acetoin	513-86-0	303.92±58.15	649.82±12.34	620.16±61.21	261.27±54.84	0±0	0±0	0±0	0±0
20.0782	3-Methylpyridine	108-99-6	0±0	0±0	0±0	0±0	0±0	230.32±81.03	0±0	0±0
22.2869	Dimethyl trisulfide	3658-80-8	0±0	348.62±23.3	778.88±52.75	1538.75±76.52	0±0	0±0	0±0	0±0
31.2501	1,1,5-Trimethyl-1,2-dihydronaphthalene	1000357-25-8	0±0	59.38±4.46	105.26±9.98	0±0	0±0	0±0	0±0	0±0

39: Jagella, T., & Grosch, W. Flavour and off-flavour compounds of black and white pepper (*Piper nigrum L.*). *Eur. Food Res. Technol.* **1999**, 209(1), 22–26. https://doi.org/10.1007/s002170050450

40: Prado, R., Hartung, A. C. M., Gastl, M., & Becker, T. Identification of potential odorant markers to monitor the aroma formation in kilned specialty malts. *Food Chem.* **2022**, 392, 133251. https://doi.org/10.1016/j.foodchem.2022.133251

41: Zhao, C.; Fan, W.; Xu, Y. Characterization of key aroma compounds in pixian broad bean paste through the molecular sensory science technique. *LWT-Food Sci. Technol.* **2021**, 148, 111743. https://doi.org/10.1016/j.lwt.2021.111743.

42: The compound thresholds used in this study were obtained from the Volatile Compounds in Food database (https://www.vcf-online.nl/VcfCompounds.cfm?volatgrp=15).

43: Van, L.J. Compilations of Odour Threshold Values in Air, Water and Other Media, 2nd ed.; Oliemans Punter & Partners, 2011.

44: Ritter, S.W.; Ensslin, S.; Gastl, M.I.; Becker, T.M. Identification of key aroma compounds of faba beans (Vicia faba) and their development during germination – A SENSOMICS approach. *Food Chem.* **2024**, 435, 137610. https://doi.org/10.1016/j.foodchem.2023.137610.

45: Sahin, B.; Schieberle, P. Characterization of the key aroma compounds in yeast dumplings by means of the Sensomics concept. *J. Agric. Food Chem.* **2019**, 67, 2973–2979. https://doi.org/10.1021/acs.jafc.9b00556.

46: Song, W.; Sun, M.; Lu, H.; Wang, S.; Wang, R.; Shang, X.; Feng, T. Variations in key aroma compounds and aroma profiles in yellow and white cultivars of *Flammulina filiformis* based on gas chromatography–mass spectrometry–Olfactometry, aroma recombination, and omission experiments coupled with odor threshold concentrations. *Foods* **2024**, 13, 684. https://doi.org/10.3390/foods13050684.

47: Yang, S.; Fu, J.; He, J.; Zhang, X.; Chai, L.-J.; Shi, J.-S.; Wang, S.; Zhang, S.; Shen, C.; Lu, Z.-M.; Xu, Z. Decoding the Qu-aroma of medium-temperature Daqu starter by volatilomics, aroma recombination, omission studies and sensory analysis. *Food Chem.* **2024**, 457, 140186. https://doi.org/10.1016/j.foodchem.2024.140186.

Table S2. Aroma-Active Compounds Identified Based on ROAV Values

Name	Threshold (μg/L)	T0	T10	T20	T40	S0	S10	S20	S40
1-Hexanol	832.1[39]	902.84	396.05	495.54	167.94	591.86	267.60	362.46	407.19
1-Octen-3-ol	0.08[40]	0.00	0.00	203.64	143.03	245.20	199.36	401.25	2428.53
[R-(R*,R*)]-2,3-Butanediol	80.25[41]	0.00	0.00	1688.33	2032.69	0.00	4138.61	3032.58	1126.61
Ethyl octanoate	13[42]	89.61	261.50	468.37	0.00	0.00	0.00	0.00	0.00
Ethyl phenylacetate	155.5[43]	0.00	1785.40	1662.59	304.40	0.00	0.00	0.00	0.00
3-Methyl-butanoic acid	18 [42]	0.00	5354.42	11987.72	8868.66	321.00	406.69	657.10	316.90
Hexanal	9.18 [42]	390.82	0.00	0.00	0.00	0.00	0.00	0.00	0.00
Nonanal	1.1 [42]	0.00	105.27	0.00	0.00	79.08	0.00	0.00	0.00
Benzaldehyde	7[44]	133.00	6124.90	8441.46	2643.91	0.00	604.23	696.06	202.91
Benzeneacetaldehyde	28[45]	0.00	10581.37	11855.39	3110.10	0.00	0.00	0.00	0.00
2-Octanone	176.78[46]	2349.13	0.00	0.00	0.00	2514.95	3197.15	13535.68	24408.53
2-Nonanone	5[42]	0.00	0.00	0.00	0.00	0.00	0.00	0.00	2757.34
2,5-Dimethyl-pyrazine	141.35[47]	83.06	309.64	1341.92	3495.73	0.00	2064.89	5888.95	5812.45
Trimethyl-Pyrazine	332[42]	388.30	2693.42	8009.96	13760.36	74.08	2212.80	6074.92	9657.87
2,6-Diethyl-pyrazine	123.11[47]	218.28	464.96	123.60	247.26	23.41	154.35	1118.11	1226.90
Tetramethyl-pyrazine	10,000[42]	4270.20	15148.92	39894.09	37465.48	804.60	12790.53	21262.45	24378.18
2-Methoxyphenol	0.84[42]	0.00	335.04	504.15	0.00	0.00	63.82	115.07	36.91
4-Vinylguaiacol	18.3l [42]	0.00	859.29	300.50	0.00	0.00	0.00	0.00	0.00
2-Pentyl-furan	90.24[47]	298.84	1534.81	2802.38	553.41	647.58	453.46	500.25	205.29
Acetoin	14[42]	303.92	649.82	620.16	261.27	0.00	0.00	0.00	0.00
Dimethyl trisulfide	0.36[42]	0.00	348.62	778.88	1538.75	0.00	0.00	0.00	0.00

Table S3. Differential Compounds Identified by PLS-DA for Discriminating among Different Daqu Samples

Name	T0	T10	T20	T40	S0	S10	S20	S40	*P*	FDR	VIP
Furfural	0.00	22.41	9.30	0.58	0.07	0.09	0.00	0.00	3.15E-14	2.17E-13	3.47
Tetramethylpyrazine	4.27	15.15	39.90	37.47	0.80	12.79	21.27	24.38	2.80E-11	1.10E-10	3.20
2-Octanone	2.35	0.00	0.00	0.00	2.51	3.20	13.54	24.41	0.000368834	0.000436255	3.11
Phenylethyl alcohol	0.44	4.68	4.72	18.28	0.55	11.66	13.60	7.19	3.42E-07	5.98E-07	3.03
2,3-Butanediol	0.00	0.00	1.69	2.03	0.00	4.14	3.03	1.13	1.61E-08	3.62E-08	2.21
Methyl palmitate	0.22	0.00	0.38	12.72	0.04	5.45	5.82	8.17	1.52E-09	4.30E-09	1.93
Trimethylpyrazine	0.39	2.69	8.01	13.76	0.07	2.22	6.07	9.66	2.31E-09	6.05E-09	1.91
Methyl phenylacetate	2.19	0.08	0.50	9.59	0.08	1.05	3.72	5.41	4.51E-06	6.44E-06	1.90
3-Methylbutanoic acid	0.00	5.36	11.99	8.87	0.32	0.41	0.66	0.32	7.16E-16	7.87E-15	1.87
Benzeneacetaldehyde	0.00	10.58	11.86	3.11	0.00	0.00	0.00	0.00	3.95E-16	4.82E-15	1.82
[S-(R,R)]-2,3-Butanediol	0.00	0.00	1.94	1.94	0.12	2.87	1.73	0.51	2.08E-07	3.81E-07	1.76
Acetic acid	0.00	7.86	8.80	4.36	0.00	0.45	0.29	0.24	2.54E-10	8.22E-10	1.51
Benzaldehyde	0.13	6.12	8.44	2.65	0.00	0.61	0.70	0.20	3.89E-14	2.51E-13	1.46
2,5-Dimethylpyrazine	0.08	0.31	1.34	3.50	0.00	2.07	5.89	5.81	3.60E-06	5.43E-06	1.45
Veratrole	0.02	2.17	1.50	1.07	0.00	1.08	3.25	3.49	0.003846992	0.004189794	1.37
2-Nonanone	0.00	0.00	0.00	0.00	0.00	0.00	0.00	2.76	2.33E-11	9.48E-11	1.31
2,6-Dimethylpyrazine	0.00	0.00	1.26	4.78	0.00	0.76	2.93	3.65	1.88E-10	6.63E-10	1.15
3-Methyl-1-butanol	0.00	0.00	1.31	0.00	0.00	0.47	0.89	0.81	0.000101037	0.000126297	1.13
1-Octen-3-ol	0.00	0.00	0.20	0.14	0.25	0.20	0.40	2.43	4.27E-10	1.34E-09	1.12
Methyl (Z,Z)-9,12-octadecadienoate	0.00	0.00	0.14	3.96	0.00	1.23	0.90	2.11	9.83E-08	1.93E-07	1.12
Methylpyrazine	0.00	0.00	0.32	2.38	0.00	0.00	0.00	0.00	3.98E-21	1.09E-19	1.11
Ethyl lactate	0.00	3.39	2.87	0.00	0.00	0.00	0.00	0.00	6.04E-12	2.66E-11	1.08
2-Hydroxybenzaldehyde	0.00	0.00	1.50	0.00	0.00	0.00	0.00	0.00	2.04E-05	2.74E-05	1.046
Benzyl alcohol	0.00	0.07	0.27	1.94	0.00	0.00	0.00	0.00	2.48E-24	2.73E-22	1.01

3-Furanmethanol	0.00	3.05	2.90	0.35	0.00	0.00	0.00	0.00	1.45E-21	5.33E-20	1.01

Table S4. Topological Properties of Microbial Co-occurrence Networks

	All		All_subnetwork_mean		All_subnetwork_median	
**Bacteria**						
Index	T	S	T	S	T	S
Number of components	81.00	20.00	26.44	6.00	20.00	5.00
Clustering coefficient	0.70	0.82	0.73	0.82	0.75	0.82
Modularity	0.45	0.33	0.31	0.34	0.29	0.35
Positive edge percentage	91.81	82.63	93.94	91.77	93.48	94.28
Edge density	0.04	0.17	0.12	0.26	0.10	0.22
Natural connectivity	0.07	0.17	0.14	0.20	0.12	0.19
Average dissimilarity	0.97	0.91	0.92	0.85	0.93	0.86
Average path length	0.97	1.39	0.64	1.37	0.62	1.37
**Fungi**						
Index	T	S	T	S	T	S
Number of components	1.00	1.00	1.31	1.75	1.00	2.00
Clustering coefficient	0.79	0.79	0.81	0.83	0.79	0.83
Modularity	0.11	0.09	0.13	0.09	0.09	0.12
Positive edge percentage	46.00	51.10	85.07	75.27	89.81	76.13
Edge density	0.52	0.49	0.57	0.59	0.56	0.53
Natural connectivity	0.18	0.20	0.30	0.35	0.29	0.32
Average dissimilarity	0.82	0.83	0.71	0.70	0.73	0.74
Average path length	1.14	1.11	1.06	1.04	1.07	1.03

Table S5. Topological Properties of Microbial Subnetworks

Sample	Number of components	Clustering coefficient	Modularity	Positive edge percentage	Edge density	Natural connectivity	Average dissimilarity	Average path length
**Bacteria**								
T_d0	52.50	0.75	0.16	90.78	0.08	0.11	0.96	0.77
T_d10	24.75	0.66	0.50	93.06	0.07	0.08	0.95	0.76
T_d20	12.75	0.76	0.29	97.66	0.19	0.19	0.87	0.51
T_d40	15.75	0.77	0.30	94.25	0.15	0.17	0.90	0.51
S_d0	2.50	0.90	0.02	70.41	0.64	0.39	0.69	1.12
S_d10	3.75	0.81	0.07	86.58	0.46	0.36	0.73	0.92
S_d20	3.25	0.82	0.20	89.78	0.40	0.33	0.76	0.94
S_d40	3.50	0.83	0.18	91.11	0.41	0.34	0.75	0.93
**Fungi**								
T_d0	2.00	0.90	0.01	62.36	0.71	0.38	0.67	1.08
T_d10	1.00	0.76	0.33	93.46	0.44	0.21	0.78	1.09
T_d20	1.00	0.77	0.11	93.36	0.54	0.30	0.71	1.07
T_d40	1.25	0.81	0.10	91.11	0.60	0.31	0.69	1.01
S_d0	1.75	0.82	0.12	65.17	0.52	0.27	0.79	1.10
S_d10	1.75	0.89	0.02	84.38	0.77	0.48	0.57	0.95
S_d20	1.75	0.85	0.08	82.42	0.58	0.39	0.68	1.08
S_d40	1.75	0.77	0.16	69.10	0.50	0.28	0.77	1.03

Table S6. Statistical analysis of enzymes derived from bacteria and fungi

EC number	Group	Slope (log2/day)	P value	BH FDR
		**Bacteria**		
EC:2.4.1.18	T	-0.145927081	0.00342702	0.005991641
EC:2.4.1.18	S	-0.069158201	0.000141324	0.000837313
EC:3.2.1.3	T	0.214353417	7.42727E-05	0.000505054
EC:3.2.1.3	S	0.103156886	0.000147761	0.000837313
EC:3.2.1.1	T	-0.019165797	0.000735212	0.002173669
EC:3.2.1.1	S	-0.020310366	0.002300677	0.004601354
EC:3.2.1.10	T	0.005323681	0.703321431	0.74727902
EC:3.2.1.10	S	0.037396433	0.018666822	0.024889096
EC:3.2.1.20	T	0.078168544	0.000566748	0.001835183
EC:3.2.1.20	S	0.03365292	0.004365988	0.00724115
EC:2.4.1.25	T	-0.242067462	0.000288265	0.001153058
EC:2.4.1.25	S	-0.141328113	0.000233378	0.001057982
EC:2.4.1.1	T	-0.031104802	0.005020715	0.007839988
EC:2.4.1.1	S	-0.05550371	0.000287728	0.001153058
EC:2.7.1.2	T	0.001622383	0.098748518	0.11592479
EC:2.7.1.2	S	0.004974486	0.098877027	0.11592479
EC:5.4.2.2	T	-0.069156881	0.000208158	0.001011054
EC:5.4.2.2	S	-0.00701518	0.005211146	0.007874621
EC:5.3.1.9	T	-0.01699146	3.10917E-05	0.000395209
EC:5.3.1.9	S	-0.003299109	0.001126084	0.00280609
EC:2.7.1.11	T	-0.018039619	0.000339734	0.001256376

EC:2.7.1.11	S	-0.005403008	0.002612608	0.005075924
EC:4.1.2.13	T	-0.013809034	3.58676E-05	0.000395209
EC:4.1.2.13	S	-0.011958842	0.003788718	0.00644082
EC:2.7.2.3	T	-0.016966778	3.38956E-05	0.000395209
EC:2.7.2.3	S	-0.003275221	0.001155449	0.00280609
EC:5.4.2.12	T	0.005094493	0.011449882	0.015920325
EC:5.4.2.12	S	-0.009723917	0.011471999	0.015920325
EC:4.2.1.11	T	-0.016953195	3.51221E-05	0.000395209
EC:4.2.1.11	S	-0.002796713	4.06833E-05	0.000395209
EC:2.7.1.40	T	-0.034761037	3.53194E-05	0.000395209
EC:2.7.1.40	S	-0.020016079	6.25353E-05	0.000473359
EC:1.2.7.1	T	-0.19062105	0.000351046	0.001256376
EC:1.2.7.1	S	-0.122136987	0.006922934	0.010233902
EC:3.1.2.21	T	-0.143875098	0.005072934	0.007839988
EC:3.1.2.21	S	0.088278792	0.00172409	0.003552671
EC:3.1.1.1	T	0.036237243	0.017381384	0.023638682
EC:3.1.1.1	S	0.048735586	0.007589873	0.010981093
EC:3.1.1.3	T	0.135584899	0.001474849	0.003134053
EC:3.1.1.3	S	0.069575585	0.001206214	0.002828364
EC:1.2.4.1	T	0.017746367	0.00018465	0.00096586
EC:1.2.4.1	S	0.013116474	0.001343466	0.002946957
EC:1.1.1.1	T	0.010328648	0.000784846	0.00222373
EC:1.1.1.1	S	-0.001478371	0.255229354	0.284517969
EC:1.1.1.90	T	0.10145181	0.029682816	0.03881599

EC:1.1.1.90	S	0.039941205	0.037599788	0.048241237
EC:1.4.3.4	T	0.162553859	0.000707258	0.002173669
EC:1.4.3.4	S	0.075466067	0.001264818	0.002866921
EC:4.1.1.28	T	0.21254136	6.26505E-05	0.000473359
EC:4.1.1.28	S	0.110109626	0.000432584	0.001470787
EC:2.2.1.6	T	0.008102765	0.001154466	0.00280609
EC:2.2.1.6	S	-0.001629465	0.075917474	0.095599783
EC:4.1.1.5	T	-0.105416653	0.004846314	0.007839988
EC:4.1.1.5	S	0.067402316	0.001112854	0.00280609
EC:1.1.1.304	T	0.02497834	0.310670884	0.335327304
EC:1.1.1.304	S	0.076793681	0.00311757	0.005729589
EC:1.1.1.303	T	-0.031984618	0.208116334	0.235865179
EC:1.1.1.303	S	0.03049777	0.084280434	0.102340527
EC:1.1.1.4	T	-0.031984618	0.208116334	0.235865179
EC:1.1.1.4	S	0.03049777	0.084280434	0.102340527
EC:1.1.1.76	T	0.02497834	0.310670884	0.335327304
EC:1.1.1.76	S	0.076793681	0.00311757	0.005729589
EC:1.14.13.1	T	0.204216737	7.1396E-06	0.000395209
EC:1.14.13.1	S	0.096882579	0.003436383	0.005991641
		**Fungi**		
EC:2.4.1.18	T	-0.005439773	0.005624894	0.00843734
EC:2.4.1.18	S	-0.003292401	0.000624167	0.001994731
EC:3.2.1.3	T	0.002282422	0.457904266	0.475515969
EC:3.2.1.3	S	-0.002300855	0.006450966	0.009414923

EC:3.2.1.1	T	0.016300544	0.005072002	0.00843734
EC:3.2.1.1	S	0.006193693	6.02504E-05	0.00095401
EC:3.2.1.10	T	-0.22951986	0.006912377	0.009822852
EC:3.2.1.10	S	0.038690715	0.001258675	0.003398424
EC:3.2.1.20	T	0.00595078	0.002615499	0.005884873
EC:3.2.1.20	S	0.001332155	0.036485143	0.04690947
EC:2.4.1.1	T	-0.00532089	0.005093884	0.00843734
EC:2.4.1.1	S	-0.003293351	0.000627971	0.001994731
EC:2.7.1.1	T	-0.002248495	0.216983537	0.244106479
EC:2.7.1.1	S	-0.000127386	0.818884695	0.834335349
EC:2.7.1.2	T	0.009806893	0.010674687	0.014780336
EC:2.7.1.2	S	0.008778292	0.001386211	0.003564544
EC:5.4.2.2	T	-0.005458814	0.005438972	0.00843734
EC:5.4.2.2	S	-0.003297695	0.000591674	0.001994731
EC:5.3.1.9	T	-0.00545619	0.005418859	0.00843734
EC:5.3.1.9	S	-0.003291955	0.000622282	0.001994731
EC:2.7.1.11	T	0.00025897	0.916305779	0.916305779
EC:2.7.1.11	S	0.005691491	0.003731459	0.007749953
EC:4.1.2.13	T	-0.006899113	0.000106001	0.00095401
EC:4.1.2.13	S	-0.00552391	0.003425389	0.00739884
EC:2.7.2.3	T	-0.00545619	0.005418859	0.00843734
EC:2.7.2.3	S	-0.003291955	0.000622282	0.001994731
EC:4.2.1.11	T	-0.00545638	0.005417967	0.00843734
EC:4.2.1.11	S	-0.003291363	0.000620953	0.001994731

EC:2.7.1.40	T	-0.00545619	0.005418859	0.00843734
EC:2.7.1.40	S	-0.003291955	0.000622282	0.001994731
EC:2.3.1.85	T	-0.005079219	0.300121322	0.324131028
EC:2.3.1.85	S	-0.011364475	0.072491975	0.091036434
EC:3.1.1.1	T	0.006219983	0.3332095	0.352810059
EC:3.1.1.1	S	0.001200178	0.098666722	0.115826152
EC:3.1.1.3	T	-0.012819905	8.32341E-05	0.00095401
EC:3.1.1.3	S	-0.009388282	0.00193874	0.004758724
EC:1.2.4.1	T	0.001853696	0.081833781	0.098200538
EC:1.2.4.1	S	-0.000122226	0.147087268	0.168993883
EC:4.1.1.1	T	0.007549257	0.004474662	0.00843734
EC:4.1.1.1	S	0.006276546	0.000378952	0.001994731
EC:1.1.1.1	T	0.007497334	0.078468263	0.096301959
EC:1.1.1.1	S	0.006601406	1.3899E-05	0.000750547
EC:1.1.1.2	T	-0.011938482	0.000224504	0.001515401
EC:1.1.1.2	S	-0.009067956	0.005542613	0.00843734
EC:1.4.3.4	T	0.013012233	0.238996039	0.26338339
EC:1.4.3.4	S	0.018305223	6.46964E-05	0.00095401
EC:4.1.1.28	T	-0.005454334	0.012824728	0.016891105
EC:4.1.1.28	S	-0.005559006	0.000789719	0.002369156
EC:1.1.1.4	T	0.022691324	0.000870349	0.002473622
EC:1.1.1.4	S	0.011775208	0.000392212	0.001994731
EC:1.14.13.1	T	0.008872852	0.011444998	0.015450747
EC:1.14.13.1	S	0.005646744	9.04731E-05	0.00095401

EC:2.1.1.6	T	0.017352718	0.00208186	0.004887845
EC:2.1.1.6	S	0.006906643	0.000148385	0.001144684

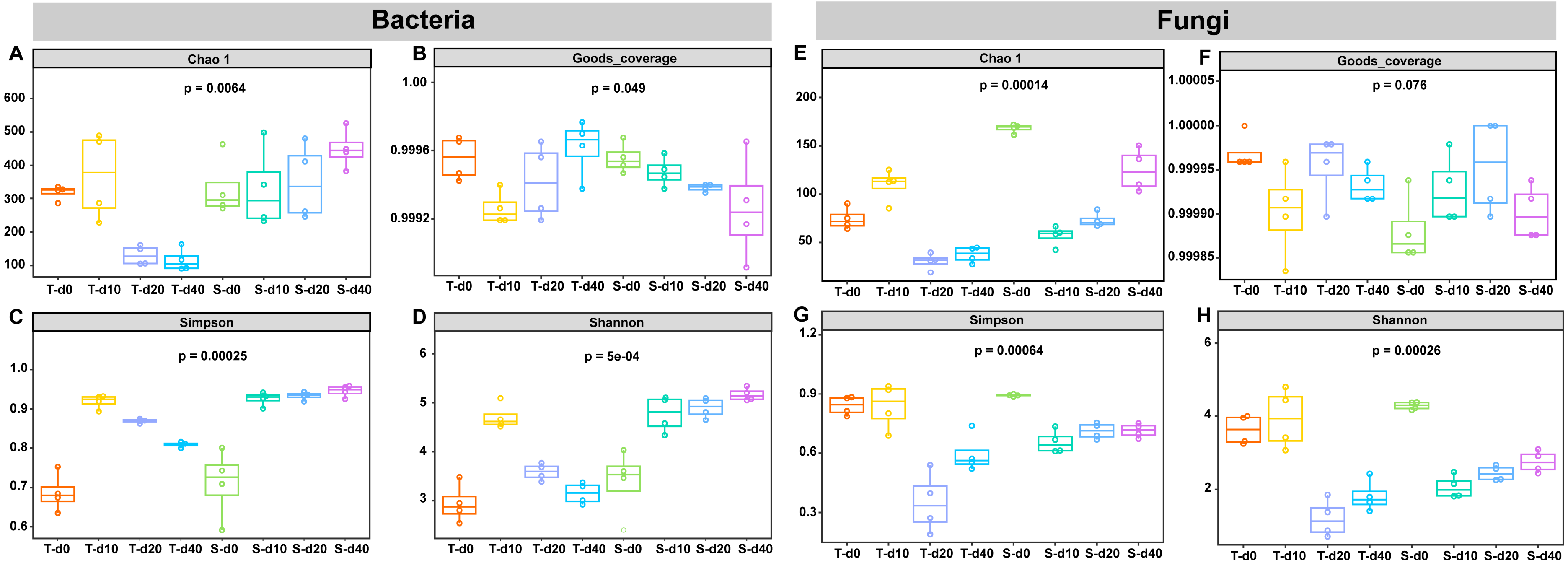

Figure S1. Alpha-diversity indices of bacterial communities at different fermentation stages (A, B, C, D). Alpha-diversity indices of fungal communities at different fermentation stages (E, F, G, H). (T represents traditional naturally fermented Daqu, S represents Daqu fermented with surface-disinfected wheat, and D0, D10, D20, and D40 represent fermentation days 0, 10, 20, and 40, respectively.)

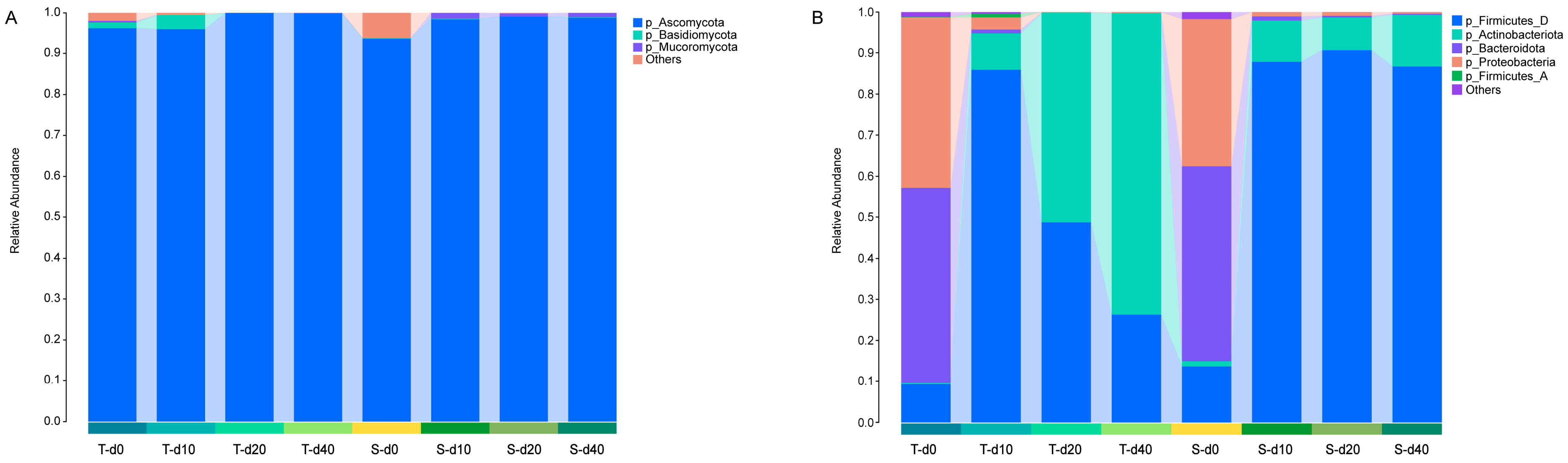

Figure S2. Microbial Community Composition at the Phylum Level during Daqu Fermentation.

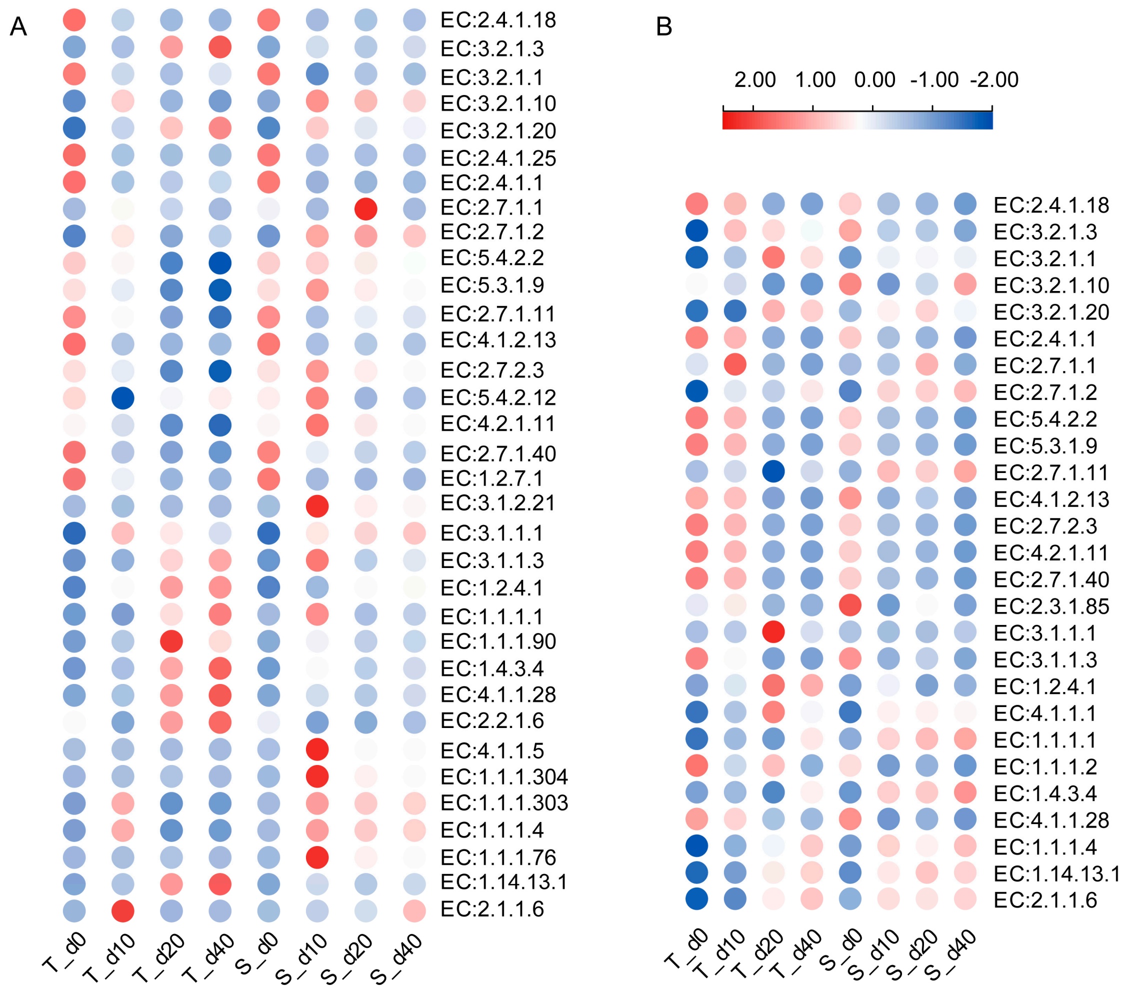

Figure S3. Abundance of Microbial Enzymes Related to Flavor Compound Formation during Daqu Fermentation. (A) Bacteria-derived enzymes; (B) fungi-derived enzymes.
